# Supplementary material for: Farnesyltransferase inhibition overcomes oncogene-addicted non-small cell lung cancer adaptive resistance to targeted therapies
Source: Nat Commun. 2024 Jun 27;15:5345. doi: 10.1038/s41467-024-49360-4 (PMC11211478; doi:10.1038/s41467-024-49360-4)

SUPPLEMENTARY INFORMATION

Title: Farnesyltransferase inhibition overcomes oncogene-addicted non-small cell lung cancer adaptive resistance to targeted therapies

Sarah Figarol[1*], Célia Delahaye[1*], Rémi Gence[1], Aurélia Doussine[1], Juan Pablo Cerapio[1], Mathylda Brachais[1], Claudine Tardy[1], Nicolas Béry[1], Raghda Asslan[1], Jacques Colinge[4], Jean-Philippe Villemin[4], Antonio Maraver[4], Irene Ferrer[5], Luis Paz-Ares[5], Linda Kessler[6], Francis Burrows[6], Isabelle Lajoie-Mazenc[1], Vincent Dongay[1,3], Clara Morin[1,3], Amélie Florent[1], Sandra Pagano[1], Estelle Taranchon Clermont[1,2], Anne Casanova[2], Anne Pradines[1,2], Julien Mazieres[1,3], Gilles Favre[1,2#*], Olivier Calvayrac[1#*]

1 Centre de Recherches en Cancérologie de Toulouse (CRCT), Inserm, CNRS, Université de Toulouse, Université Toulouse III Paul Sabatier, Toulouse, France
2 Oncopole Claudius Regaud, Institut Universitaire du Cancer de Toulouse-Oncopole, Laboratoire de Biologie Médicale Oncologique, Toulouse, France
3 Centre Hospitalier Universitaire (CHU) de Toulouse, service de pneumologie, Toulouse, France
4 Institut de Recherche en Cancérologie de Montpellier (IRCM), Inserm, Université de Montpellier, Institut Régional du Cancer de Montpellier (ICM), Montpellier, France
5 Unidad de Investigación Clínica de Cáncer de Pulmón, Instituto de Investigación Hospital 12 de Octubre-CNIO, Madrid, Spain
6 Kura Oncology, Inc., San Diego, USA

* Equal contribution
# Corresponding authors: olivier.calvayrac@inserm.fr; favre.gilles@iuct-oncopole.fr

A

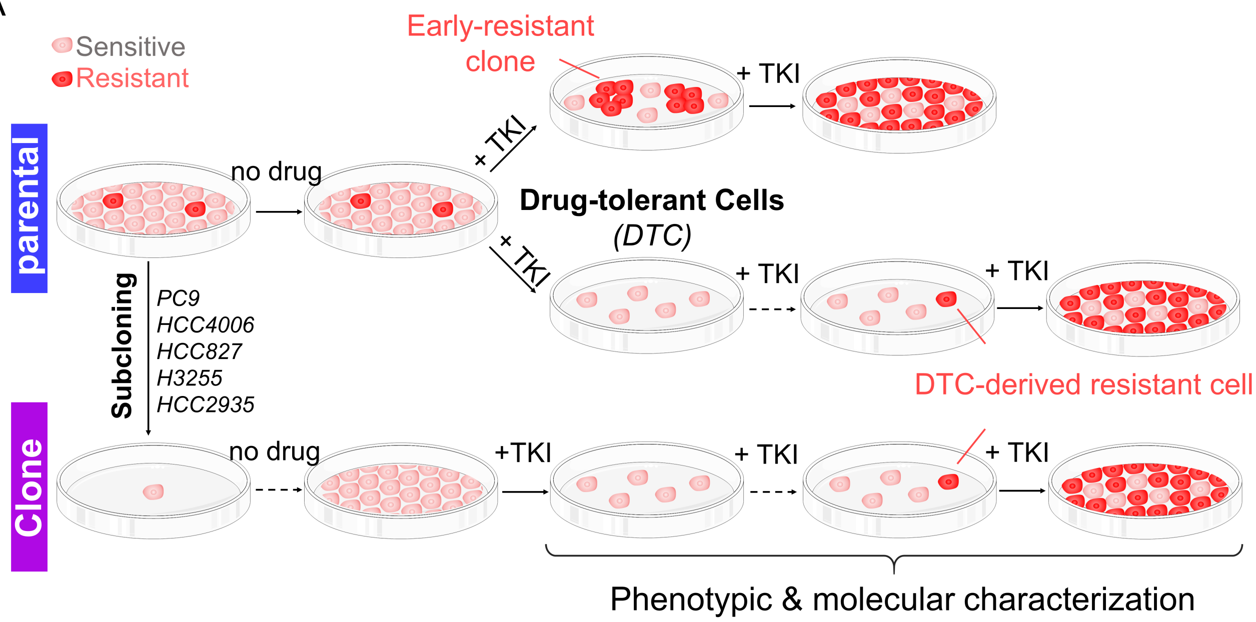

B

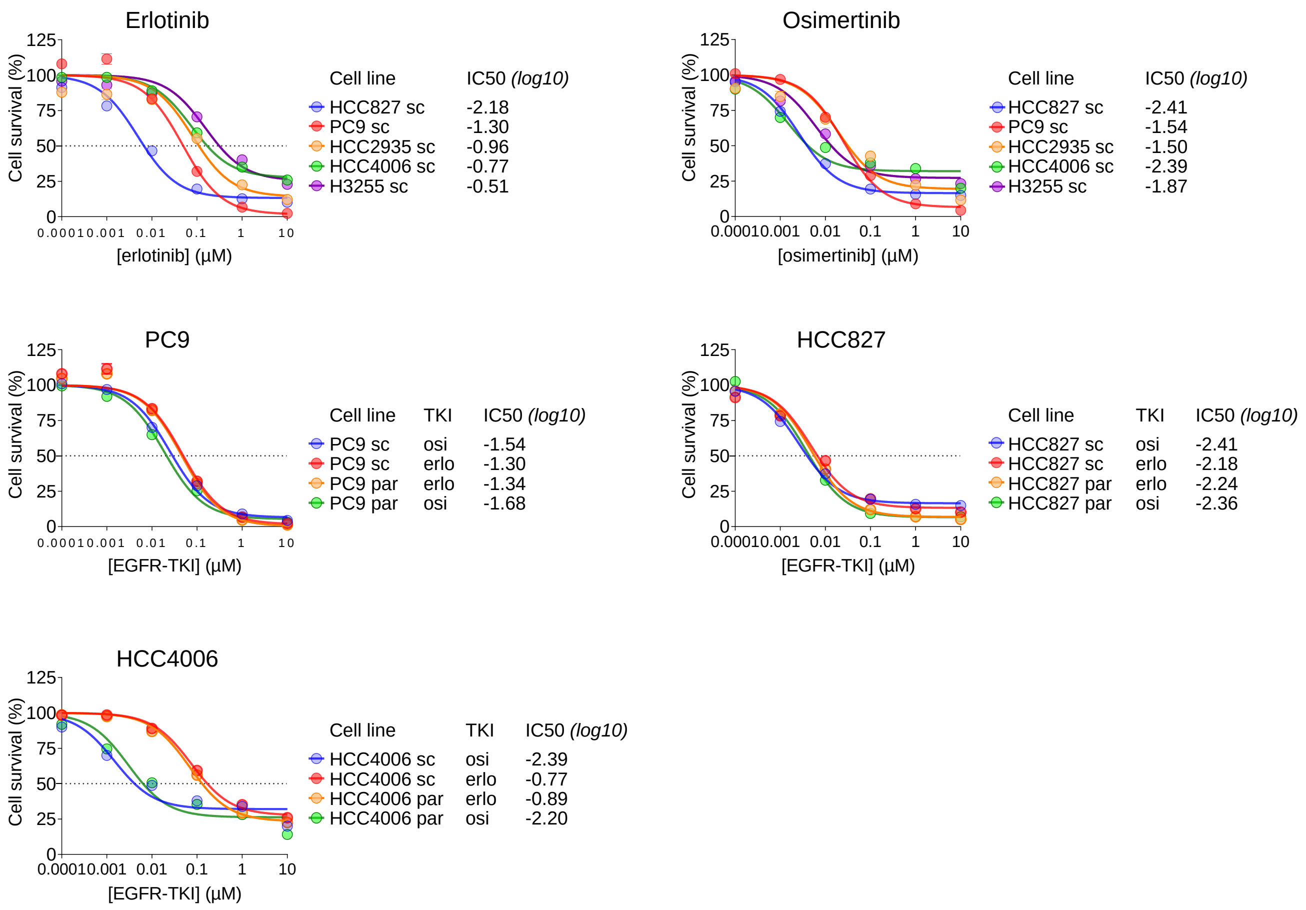

Supplementary Figure 1: Generation and characterization of clonal EGFR-mutant cell lines

A: Experimental design. Parental EGFR-mutated PC9, HCC4006, HCC827, H3255 and HCC2935 cells were subcloned in order to avoid/minimize the presence of potential pre-existing EGFR-TKI-resistant cells. Then DTC and RPC (Resistant Proliferative Cell) were characterized at phenotypic and molecular level. TKI: Tyrosine Kinase Inhibitor.

B: Cell survival (%) by cytotoxicity assay of parental (par) and subclonal (sc) PC9, HCC4006, HCC827, H3255 and HCC2935 cells upon 1 µM erlotinib or osimertinib treatment for 5 days. Half inhibitory concentration for each condition were evaluated (log10). Representative data from *n*=3 independent biological experiments.

Source data are provided as a Source data file.

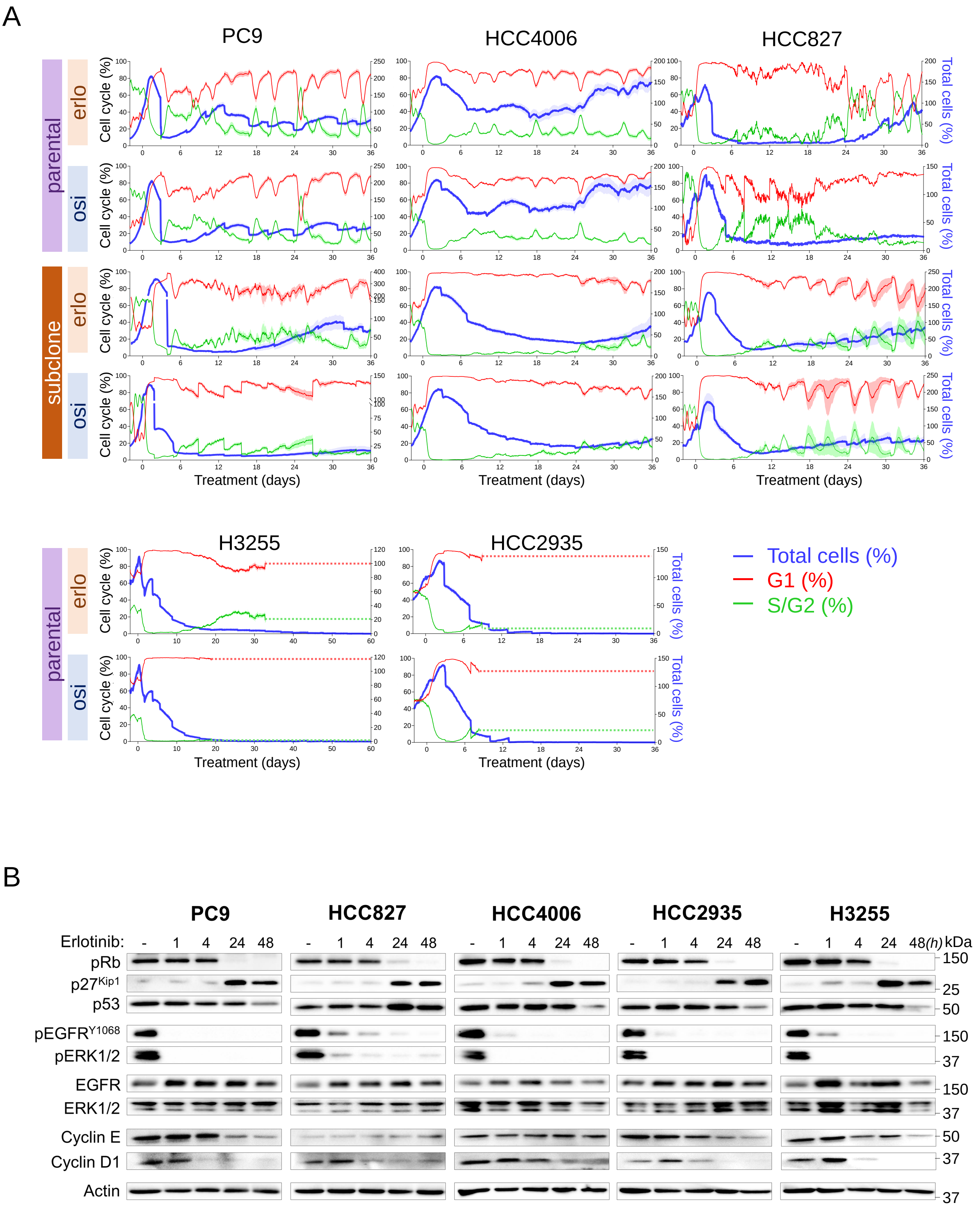

Supplementary Figure 2: Response kinetics to EGFR-Tyrosine Kinase Inhibitors in parental and clonal *EGFR*-mutant cell lines

A: Proportion of tumor cells (blue) and cell cycle dynamics (red: G1, green: S/G2) of HCC4006, PC9, HCC827, H3255 and HCC2935 parental (par) or subclonal (sc) cells during erlotinib or osimertinib treatment (1 µM). Data are mean ± SEM. Representative data from *n*=4 independent biological experiments.

B: Western blot analysis of proteins related to EGFR pathway (phospho-EGFR, EGFR, phospho-ERK, ERK) and cell cycle (p27Kip1, p53, phospho-RB, RB, cyclin E, cyclin D1) in PC9, HCC4006, HCC827, HCC2935 and H3255 cells treated with erlotinib (1 µM) at the indicated times. Representative blots from *n*=3 independent biological experiments. Source data are provided as a Source data file.

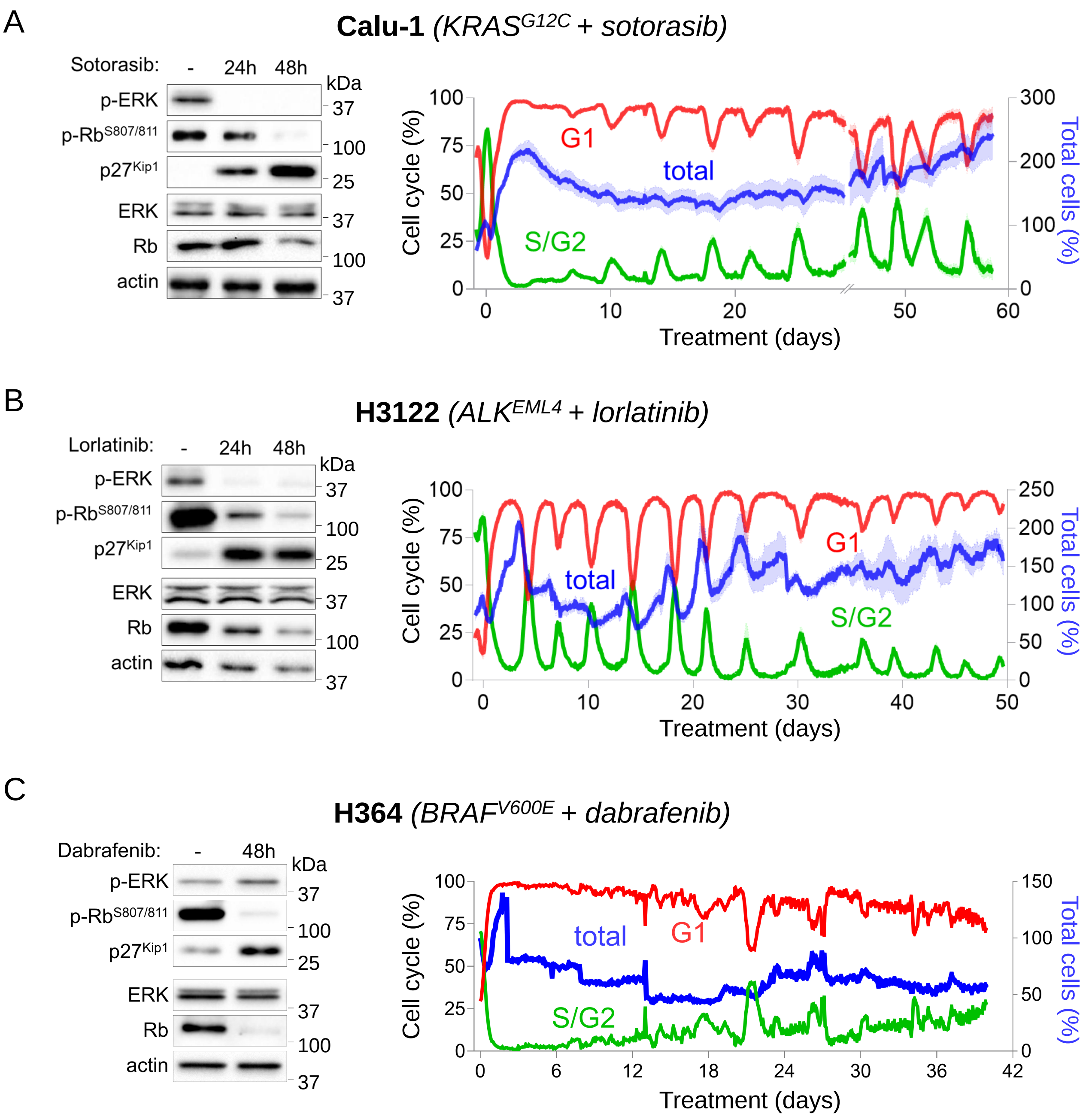

Supplementary Figure 3: Response kinetics to targeted therapies in KRAS^{G12C}, ALK^{EML4} and BRAF^{V600E} models
Left: Western blot analysis of proteins related to cell proliferation. Right: percentage of total tumor cells (blue) and cell cycle dynamics (red: G1, green: S/G2) of Calu-1 (**A**), H3122 (**B**), H364 (**C**) cells during sotorasib, lorlatinib and dabrafenib treatment respectively (1 µM). Data shown in the graphs on the right are mean ± SEM. Blots are representative from *n*=3 independent biological experiments. Incucyte analyses are representative from *n*=3 (Calu-1) or *n*=2 (H3122 and HCC364) independent biological experiments.
Source data are provided as a Source data file.

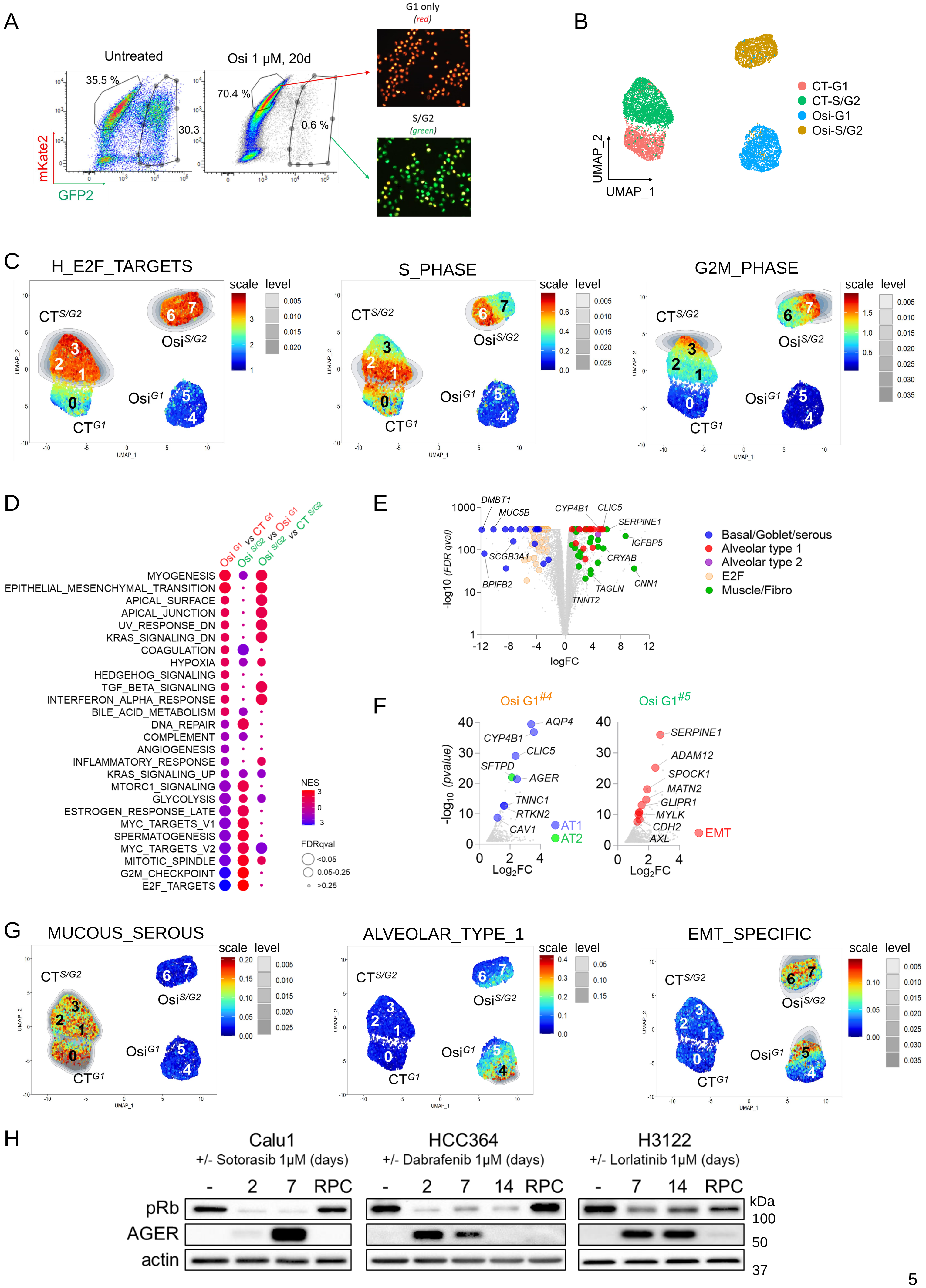
A
Untreated
Osi 1 µM, 20d
35.5 %
30.3
70.4 %
0.6 %
mKate2
GFP2
G1 only (red)
S/G2 (green)
B
CT-G1
CT-S/G2
Osi-G1
Osi-S/G2
UMAP_1
UMAP_2
C
H_E2F_TARGETS
S_PHASE
G2M_PHASE
scale
level
D
Osi G1 vs CT G1
Osi S/G2 vs Osi G1
Osi S/G2 vs CT S/G2
MYOGENESIS
EPITHELIAL_MESENCHYMAL_TRANSITION
APICAL_SURFACE
APICAL_JUNCTION
UV_RESPONSE_DN
KRAS_SIGNALING_DN
COAGULATION
HYPOXIA
HEDGEHOG_SIGNALING
TGF_BETA_SIGNALING
INTERFERON_ALPHA_RESPONSE
BILE_ACID_METABOLISM
DNA_REPAIR
COMPLEMENT
ANGIOGENESIS
INFLAMMATORY_RESPONSE
KRAS_SIGNALING_UP
MTORC1_SIGNALING
GLYCOLYSIS
ESTROGEN_RESPONSE_LATE
MYC_TARGETS_V1
SPERMATOGENESIS
MYC_TARGETS_V2
MITOTIC_SPINDLE
G2M_CHECKPOINT
E2F_TARGETS
NES
FDRqval
<0.05
0.05-0.25
>0.25
E
-log10 (FDR qval)
logFC
DMBT1
MUC5B
CYP4B1
CLIC5
SERPINE1
IGFBP5
SCGB3A1
CRYAB
BPIFB2
TAGLN
CNN1
TNNT2
Basal/Goblet/serous
Alveolar type 1
Alveolar type 2
E2F
Muscle/Fibro
F
Osi G1#4
Osi G1#5
-log10 (pvalue)
Log2FC
AQP4
CYP4B1
CLIC5
SFTPD
AGER
TNNC1
RTKN2
CAV1
AT1
AT2
SERPINE1
ADAM12
SPOCK1
MATN2
GLIPR1
MYLK
CDH2
AXL
EMT
G
MUCOUS_SEROUS
ALVEOLAR_TYPE_1
EMT_SPECIFIC
H
Calu1
+/- Sotorasib 1µM (days)
HCC364
+/- Dabrafenib 1µM (days)
H3122
+/- Lorlatinib 1µM (days)
RPC
pRb
AGER
actin
kDa
100
50
37

Supplementary Figure 4: Characterization of drug-tolerant subpopulations identified by single-cell RNA-seq

A: Flow cytometry charts of red cells (G1) or green cells (S/G2 or G1-S) of untreated or osimertinib treated (1 µM, 20 days) HCC4006 subclonal cells sorted for single-cell RNA sequencing experiment. Percentages of each fraction according to total population are specified.

B: UMAP representation of the cells clustering obtained after scRNAseq. Cells are colored based on their treatment and cell cycle phase. CT: Control; Osi: osimertinib.

C: Distribution of signature score of E2F_targets, S-phase or G2M-phase in the different clusters. Gene signatures for S and G2M phases are from Itay Tirosh *et al.*[1].

D: GSEA of Hallmark gene sets comparing osimertinib-treated G1 *vs* untreated-G1 HCC4006 cells, osimertinib-treated S/G2 *vs* osimertinib-treated G1 HCC4006 cells or osimertinib-treated S/G2 *versus* untreated S/G2 HCC4006 cells.

E: Volcano plot of the differentially expressed genes between osimertinib-G1 and untreated-G1 HCC4006 clonal cells

F: Osimertinib-induced genes overexpressed specifically in G1 cluster 4 *vs* cluster 5 (left) or in cluster 5 *vs* cluster 4 (right). AT1 (blue), AT2 (green) or EMT (red)-specific genes are highlighted. AT: Alveolar Type

G: Distribution of signature score of mucous/serous, alveolar type 1 and EMT-specific signatures in the different clusters

H: Western blot analysis of phosphorylated Rb and AGER during drug-tolerance and after relapse in $KRAS^{G12C}$ (Calu-1), $BRAF^{V600E}$ (HCC364) and ALK^{EML4} (H3122)-mutant cell lines treated respectively with sotorasib, dabrafenib or lorlatinib at 1µM. RPC: Resistant Proliferative Cells. Blots are representative from *n*=3 independent biological experiments.

Source data are provided as a Source data file.

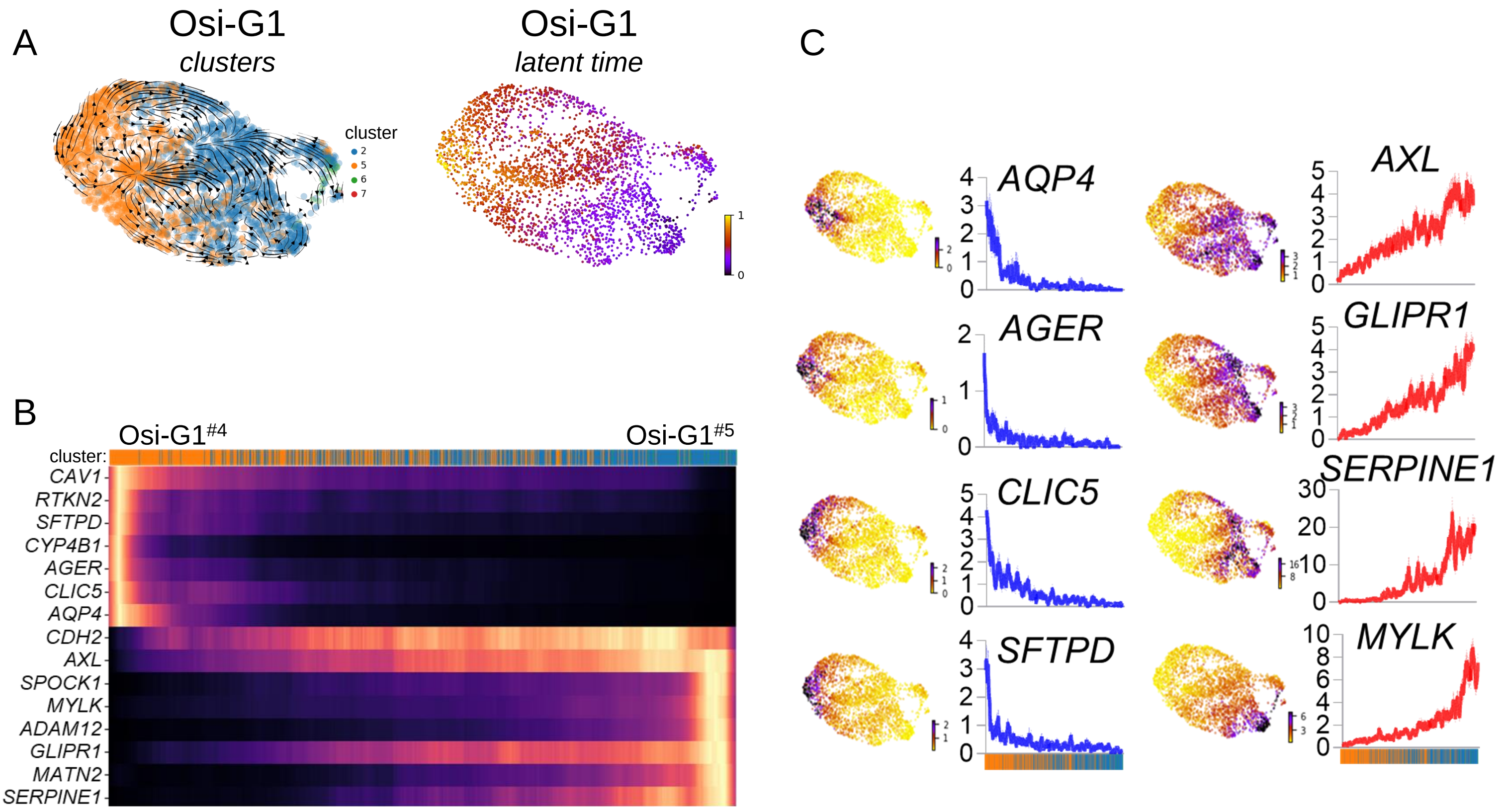

Supplementary Figure 5: Pseudotime analysis of osmertinib-treated G1 subpopulation

A: Velocity and clustering (left) and latent time (right) analysis of the osimertinib-treated G1 subpopulation.

B: Normalized mRNA expression of alveolar and EMT-related genes according to latent time in the osimertinib-treated G1 subpopulation.

C: Distribution of AT1- and EMT-related genes expression within the osimertinib-treated G1 cluster and z-score normalized mRNA expression of corresponding genes according to the latent time. Data are mean ± SEM. Orange: Osi-G1#4O, bleu: Osi-G1#5 cluster.

Source data are provided as a Source data file.

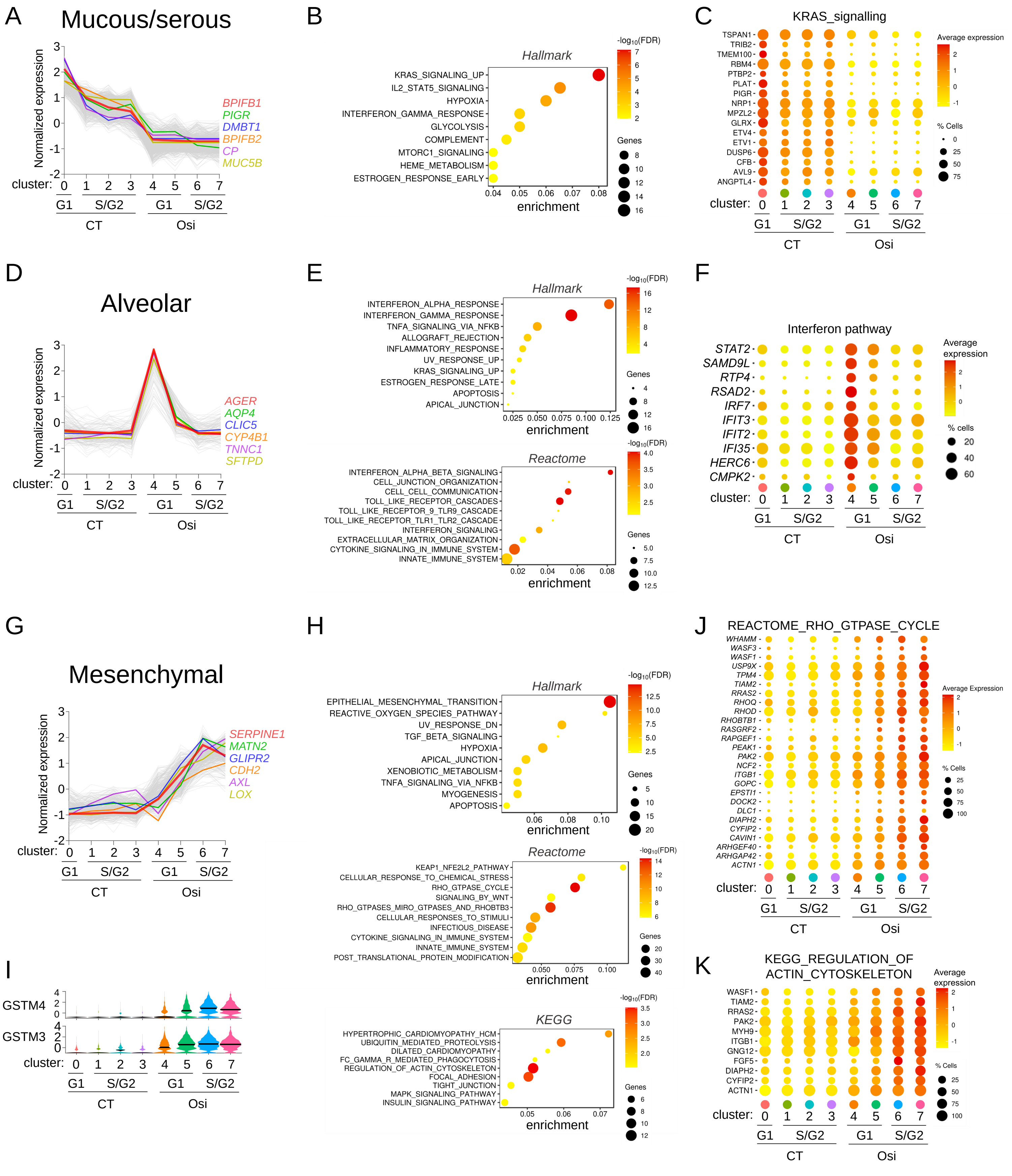

Supplementary Figure 6: Correlation analysis of genes that are co-expressed in the different phenotypes

A, D, G: Mean expression profile of genes that positively correlated (pearson correlation coefficient >0.9) with *BPIFB1* (**A**), *AGER* (**D**), and *SERPINE1* (**G**).

B, E, H: Dot plot of the top gene signatures associated with the mucous/serous (**B**), alveolar (**E**), and mesenchymal (**H**)-related genes.

C, F, J, K: Dot plot of gene expression associated with KRAS_signaling (**C**), interferon (**F**), Rho_GTPase_cycle (**J**) and actin_cytoskeleton (**K**) signatures.

I: Distribution of expression levels of glutathione-related genes.

Source data are provided as a Source data file.

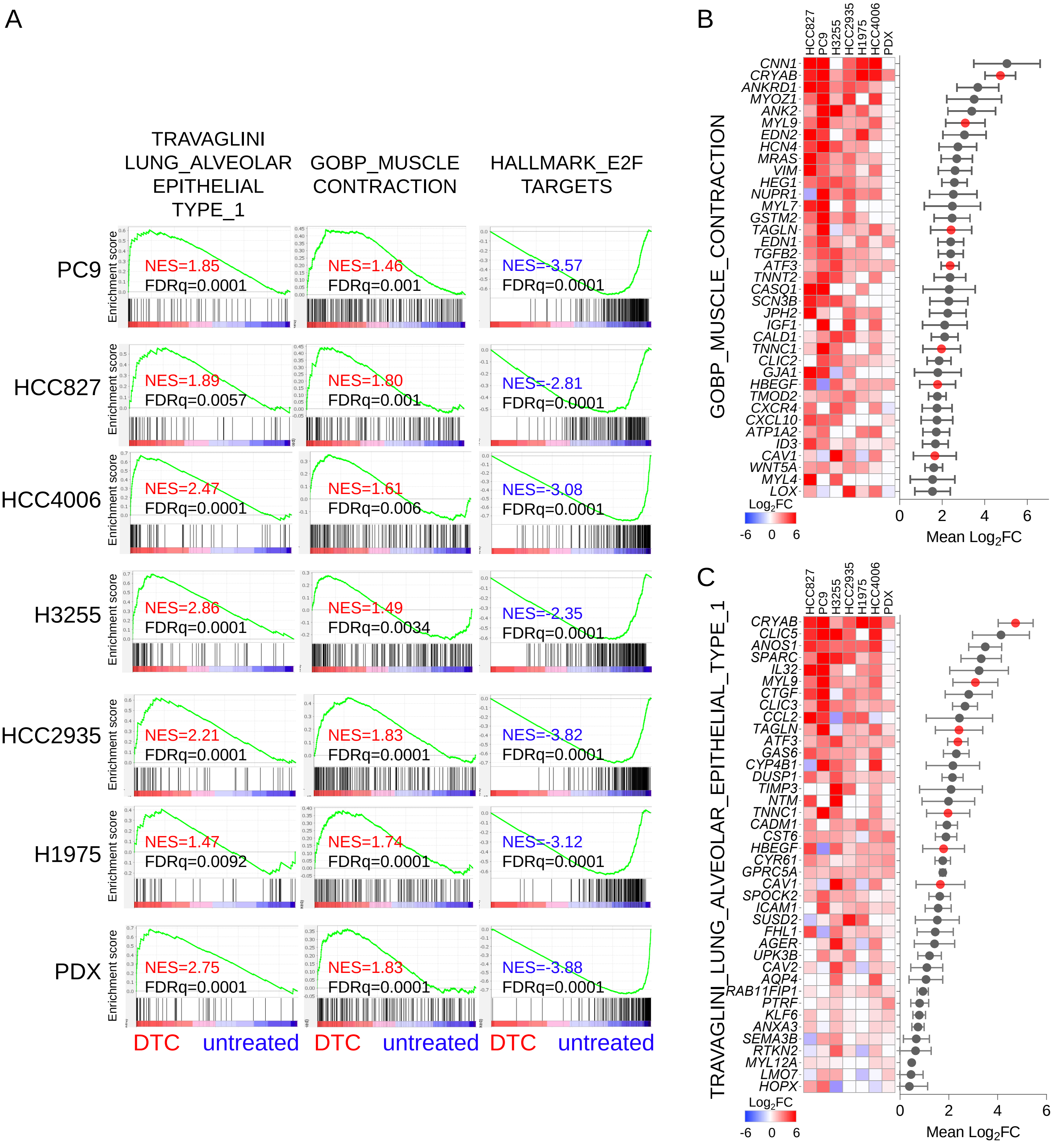

Supplementary Figure 7: Transcriptomic signatures of EGFR-TKI treated drug-tolerant cells in different EGFR-mutant models

A: GSEA analyses of TRAVAGLINI_LUNG_ALVEOLAR_EPITHELIAL TYPE_1, GOBP_MUSCLE_CONTRACTION and HALLMARK_E2F_TARGETS signatures in EGFR-mutant cell lines and PDX at DTC stage *vs* untreated.

B-C: Expression of genes involved in the GOBP_MUSCLE_CONTRACTION signature (**B**) and TRAVAGLINI_LUNG_ALVEOLAR_EPITHELIAL TYPE_1 signature (**C**) in DTC *vs* untreated in EGFR-mutant cells lines and PDX. Data are mean ±SEM. Red dots represent genes involved in both signatures. PDX EGFR$^{exon19\ \Delta E746_E750}$ transcriptomic data from Moghal *et al.*[2], GSE198672, HCC2935 and H1975 transcriptomic data from Criscione *et al.*[3], GSE193259.

Source data are provided as a Source data file.

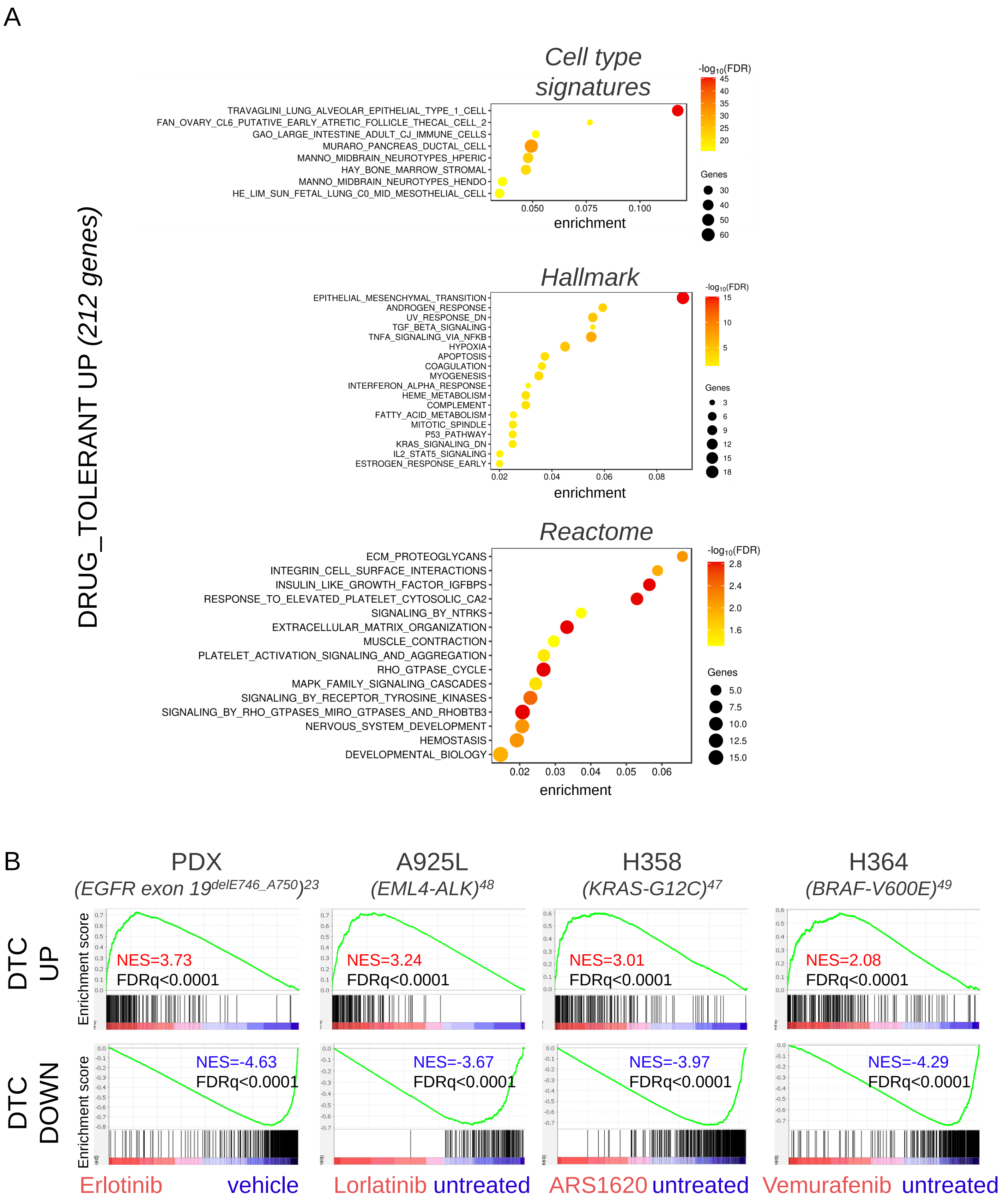

Supplementary Figure 8: Pathways associated with the drug-tolerant UP signature

A: Dot plot of the top gene signatures associated with the DTC-related genes.

B: GSEA analyses using DTC_UP and DTC_DOWN signatures in DTC generated in a PDX model of EGFR-mutant NSCLC treated until minimal residual disease with erlotinib (PDX EGFR$^{exon19\ \Delta E746_E750}$; transcriptomic data from Moghal *et al.*[2]; GSE198672, A925L cells (ALK^{EML4}; transcriptomic data from Tanimura *et al.*[4]; GSE188406), H358 cells (KRAS^{G12C}; transcriptomic data from Liu *et al.*[5]; GSE164326) and HCC364 cells (BRAF^{V600E}; transcriptomic data from Lin *et al.*[6]; GSE64550), treated with erlotinib, lorlatinib, KRASi ARS1620, and vemurafenib, respectively.

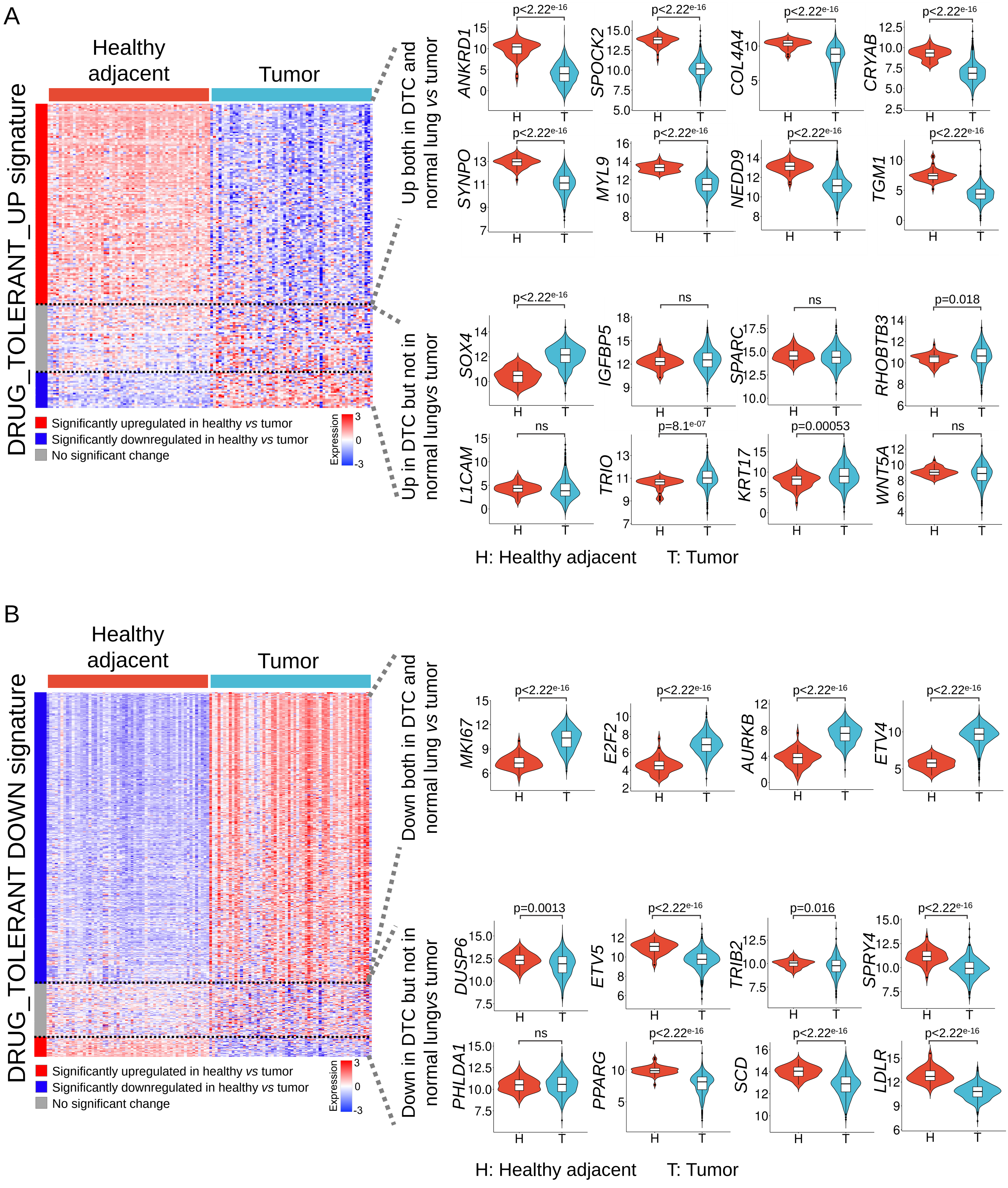

A
Healthy adjacent
Tumor
DRUG_TOLERANT_UP signature
Significantly upregulated in healthy vs tumor
Significantly downregulated in healthy vs tumor
No significant change
Expression
3
0
-3
Up both in DTC and normal lung vs tumor
Up in DTC but not in normal lungvs tumor
ANKRD1
SPOCK2
COL4A4
CRYAB
SYNPO
MYL9
NEDD9
TGM1
SOX4
IGFBP5
SPARC
RHOBTB3
L1CAM
TRIO
KRT17
WNT5A
p<2.22e-16
ns
p=0.018
p=8.1e-07
p=0.00053
H
T
H: Healthy adjacent
T: Tumor
B
Healthy adjacent
Tumor
DRUG_TOLERANT DOWN signature
Significantly upregulated in healthy vs tumor
Significantly downregulated in healthy vs tumor
No significant change
Expression
Down both in DTC and normal lung vs tumor
Down in DTC but not in normal lungvs tumor
MKI67
E2F2
AURKB
ETV4
DUSP6
ETV5
TRIB2
SPRY4
PHLDA1
PPARG
SCD
LDLR
p<2.22e-16
p=0.0013
p=0.016
ns
H: Healthy adjacent
T: Tumor

Supplementary Figure 9: Expression of genes from the drug tolerance signature in lung adenocarcinoma and healthy adjacent tissue

A: Left: Expression of genes (z-score) from the DTC_UP signature in lung adenocarcinoma (tumor, *n*=58) and healthy adjacent lung tissue (*n*=58). Right: Violin plot representing the expression of genes that are commonly upregulated in DTC and healthy lung (up) or specifically upregulated in DTC but not in healthy lung *vs* tumor (down).

B: Left: Expression of genes (z-score) from the DTC_DOWN signature in lung adenocarcinoma (tumor) and healthy adjacent lung tissue. Right: Violin plot representing the expression of genes that are commonly downregulated in DTC and healthy lung (up) or specifically downregulated in DTC but not in healthy lung *vs* tumor (down).

For **A** and **B**: Data from The Cancer Genome Atlas (TCGA) database, downloaded from http://firebrowse.org. The box plots within violin plos display 25th (lower bound), 50th (center, median), and 75th (upper bound) percentiles, with whiskers generated with the Tukey method. *p*-value was calculated using two-sided Wilcoxon test. n.s.: not significant.

Source data are provided as a Source data file.

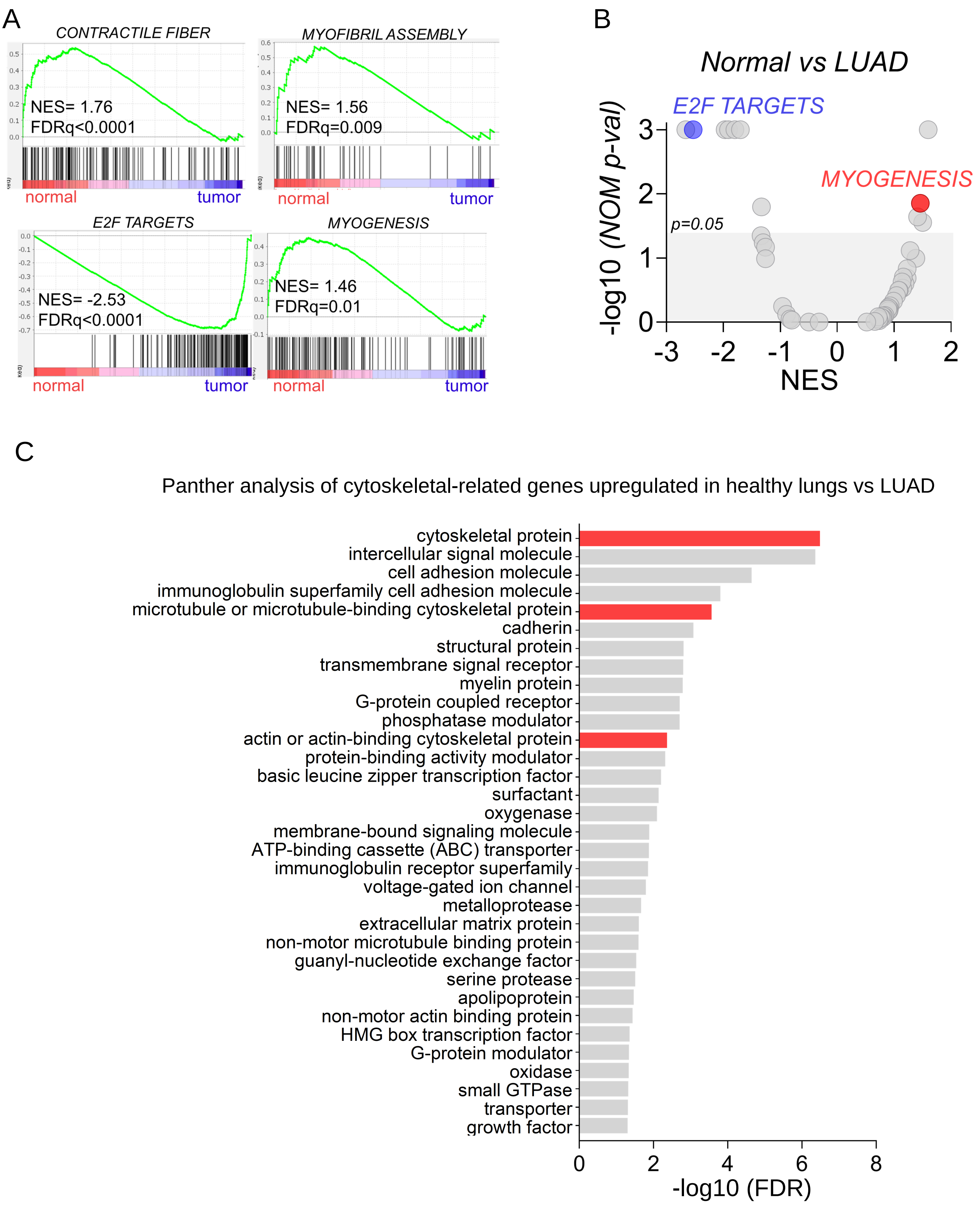

Supplementary Figure 10: Hallmarks of drug-tolerance are enriched in normal lung tissue compared to lung adenocarcinomas

A: GSEA analysis of contractile fiber, myofibril assembly, E2F_targets and myogenesis signatures in healthy lungs (*n*=58) *vs* lung adenocarcinoma (*n*=58).

B: Volcano plot of the differentially regulated gene signatures between healthy lungs *vs* lung adenocarcinoma revealed by GSEA analysis (Hallmarks and C5).

C: Top significantly over-represented protein classes (pantherbd.org) in healthy lungs *vs* lung adenocarcinoma (LUAD) (log2FC>1, *p*-value<0.05, calculated using two-tailed paired t-test.) using TCGA database.

For **A** to **C**: Data from The Cancer Genome Atlas (TCGA) database, downloaded from http://firebrowse.org.

Source data are provided as a Source data file.

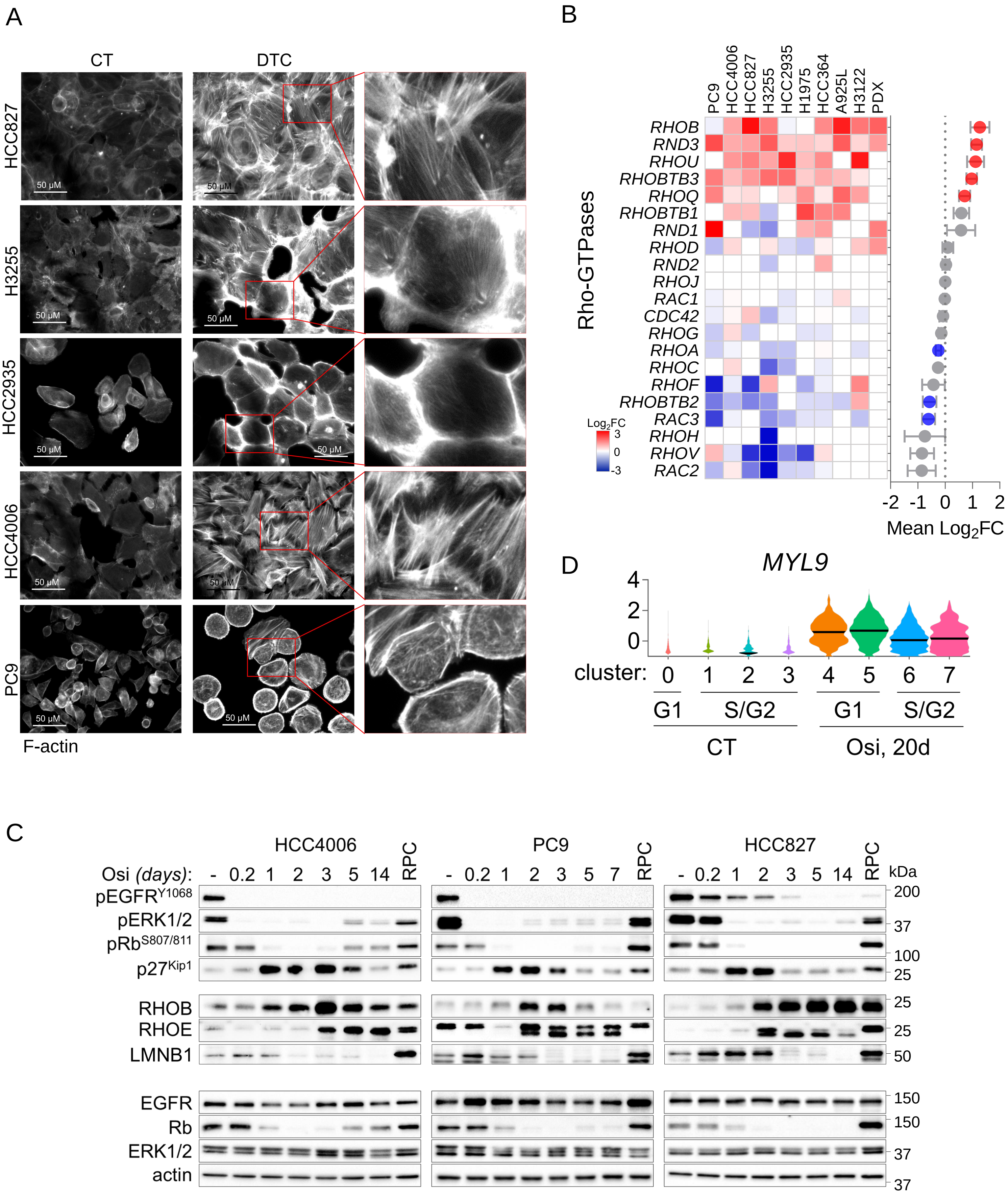
A
CT
DTC
HCC827
H3255
HCC2935
HCC4006
PC9
50 μM
F-actin
B
PC9
HCC4006
HCC827
H3255
HCC2935
H1975
HCC364
A925L
H3122
PDX
Rho-GTPases
RHOB
RND3
RHOU
RHOBTB3
RHOQ
RHOBTB1
RND1
RHOD
RND2
RHOJ
RAC1
CDC42
RHOG
RHOA
RHOC
RHOF
RHOBTB2
RAC3
RHOH
RHOV
RAC2
Log2FC
3
0
-3
-2 -1 0 1 2
Mean Log2FC
D
MYL9
4
2
0
cluster: 0 1 2 3 4 5 6 7
G1 S/G2 G1 S/G2
CT
Osi, 20d
C
HCC4006
PC9
HCC827
Osi (days): - 0.2 1 2 3 5 14 RPC
- 0.2 1 2 3 5 7 RPC
- 0.2 1 2 3 5 14 RPC
kDa
pEGFRY1068
pERK1/2
pRbS807/811
p27Kip1
RHOB
RHOE
LMNB1
EGFR
Rb
ERK1/2
actin
200
37
100
25
25
25
50
150
150
37
37

Supplementary Figure 11: Contractile phenotype and Rho-GTPases in EGFR-TKI treated cell lines

A: Phalloidin F-actin staining of HCC827, H3255, HCC2935, HCC4006 and PC9 control (CT) cells or treated cells with osimertinib (1 µM) until DTC state. Scale bar: 50µm. Representative images from *n*=5 independent biological experiments.

B: Left: differential mRNA expression of the 21 known Rho-GTPases in DTC generated from EGFR-mutant cell lines (PC9, HCC4006, HCC827, H3255, HCC2935, H1975) or PDX, $BRAF^{V600E}$ cell line (HCC364) and ALK^{EML4} cell lines (A925L, H3122) treated with appropriate targeted therapy. Right: mean Log2 fold change ±SEM. Red dots: upregulated with $p<0.01$, blue dots: downregulated with $p<0.01$, grey: non-significant. *p*-value for each individual models was obtained by DESeq2 analysis, *p*-value for mean Log2FC was calculated using two-tailed unpaired *t*-test. PDX $EGFR^{exon19\ \Delta E746\_E750}$ transcriptomic data from Moghal *et al*.[2], GSE198672; A925L cells (ALK^{EML4}) transcriptomic data from Tanimura *et al*.[4], GSE188406; H358 cells ($KRAS^{G12C}$) transcriptomic data from Liu *et al.*[5], GSE164326; HCC364 cells ($BRAF^{V600E}$) transcriptomic data from Lin *et al.*[6], GSE64550, HCC2935 and H1975 transcriptomic data from Criscione *et al*.[3], GSE193259.

C: Western Blot analysis of RHOB, RHOE/RND3, Lamin B1 (LMNB1), p27Kip1, total and phosphorylated Rb, ERK1/2 and EGFR in osimertinib-treated (1 µM) HCC4006, PC9 and HCC827 cells. Representative blots from *n*=3 independent biological experiments.

D: Distribution of normalized expression levels (z-score) of *MYL9* in untreated or osimertinib-treated G1 and S/G2 HCC4006 .

Source data are provided as a Source data file.

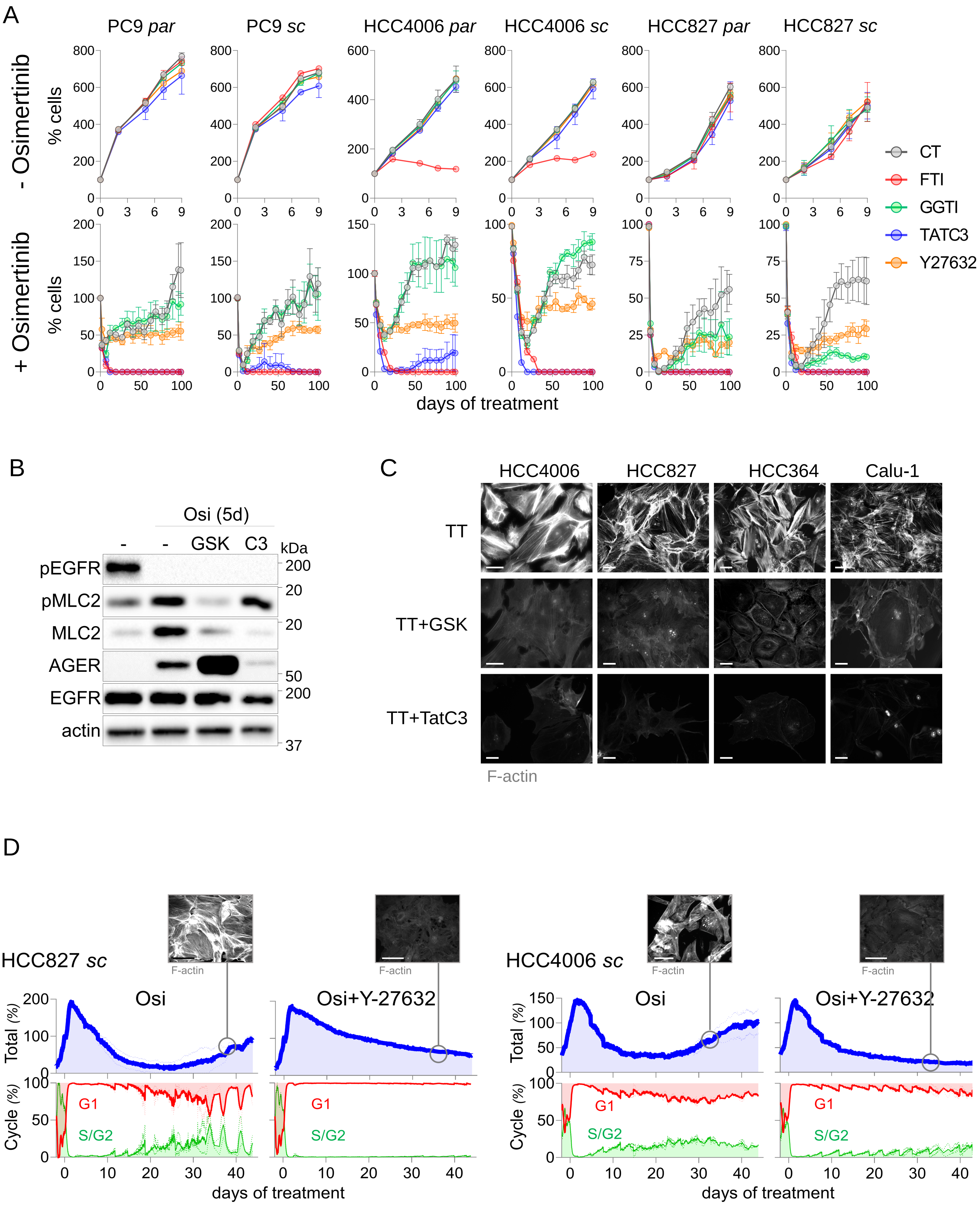
A
PC9 par
PC9 sc
HCC4006 par
HCC4006 sc
HCC827 par
HCC827 sc
- Osimertinib
+ Osimertinib
% cells
CT
FTI
GGTI
TATC3
Y27632
days of treatment
B
Osi (5d)
-
-
GSK
C3
kDa
pEGFR
pMLC2
MLC2
AGER
EGFR
actin
200
20
20
50
200
37
C
HCC4006
HCC827
HCC364
Calu-1
TT
TT+GSK
TT+TatC3
F-actin
D
HCC827 sc
HCC4006 sc
F-actin
Osi
Osi+Y-27632
Total (%)
Cycle (%)
G1
S/G2
days of treatment

Supplementary Figure 12: Response to Rho/ROCK pathway inhibitors in association with targeted therapies

A: Cell proliferation in a panel of parental and clonal population of EGFR-mutant NSCLC cell lines treated or not with farnesyltransferase inhibitor (FTI, tipifarnib, 0.1 µM), geranylgeranyltransferase inhibitor (GGTi, GGTi-298, 5 µM), ROCK1/2 inhibitor (Y27632, 5 µM) or RhoA/B/C inhibitor C3-exoenzyme (tat-C3, 5 µg/ml), alone (up) or in combination with osimertinib at 1 µM (down). Representative data from *n*=3 independent biological experiments.

B: Western blot analysis of proteins related to actomyosin contractility (MLC2, pMLC2) and alveolar type 1 phenotype (AGER) in HCC4006 subclones treated with osimertinib (1 µM) or not, alone or in combination with ROCK inhibitor (GSK269962A 5 µM), RHOA/B/C inhibitor (TatC3 10 µg/mL) or farnesyl transferase inhibitor (tipifarnib 1 µM). Representative blots from *n*=4 independent biological experiments.

C: Phalloidin F-actin staining of HCC4006, HCC827, HCC364 and Calu-1 cell lines treated with their respected targeted therapy (TT) (HCC4006 and HCC827: osimertinib 1 µM , HCC364: dabrafenib 1 µM, Calu-1: sotorasib 1 µM), alone or in combination with ROCK inhibitor (GSK269962A 5 µM), or RHOA/B/C inhibitor (TatC3 10 µg/mL). Scale bar: 20 µm. Representative images from *n*=4 independent biological experiments.

D: Incucyte monitoring showing the proportion of total (blue) G1 (red) and S/G2 (green) HCC827sc (left) or HCC4006sc (right) cells in response to 1 µM osimertinib alone or in combination with the ROCK1/2 inhibitor Y27632 at 5 µM. F-actin staining is shown. Scale bar: 50 µm

Data shown in **A** and **D** are mean ± SEM. Representative data from *n*=3 independent biological experiments. Source data are provided as a Source data file.

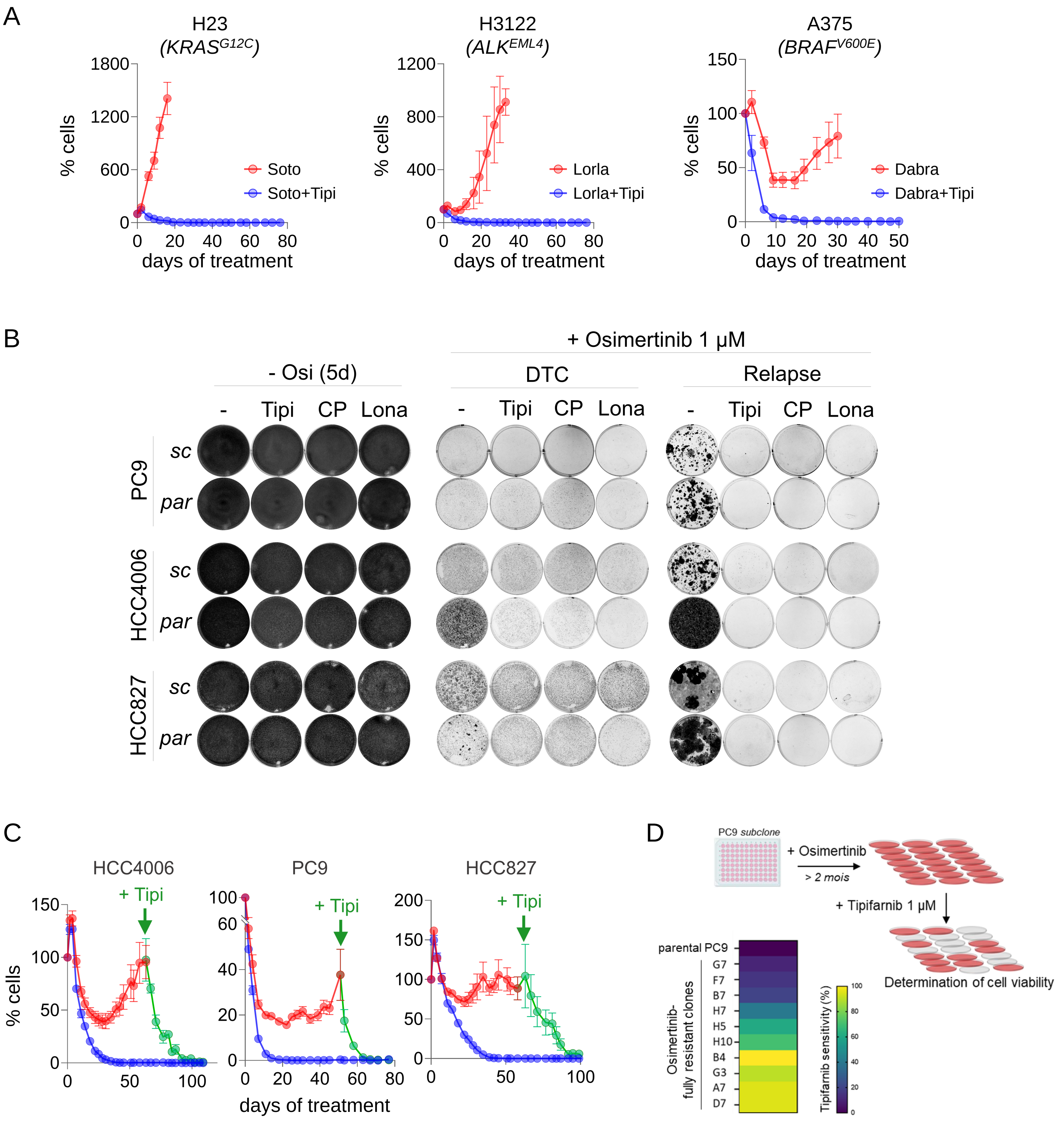
A
H23
(KRAS G12C)
1800
1200
600
0
% cells
0 20 40 60 80
days of treatment
Soto
Soto+Tipi
H3122
(ALK EML4)
1200
800
400
0
% cells
0 20 40 60 80
days of treatment
Lorla
Lorla+Tipi
A375
(BRAF V600E)
150
100
50
0
% cells
0 10 20 30 40 50
days of treatment
Dabra
Dabra+Tipi
B
+ Osimertinib 1 µM
- Osi (5d)
DTC
Relapse
- Tipi CP Lona
- Tipi CP Lona
- Tipi CP Lona
PC9
sc
par
HCC4006
sc
par
HCC827
sc
par
C
HCC4006
+ Tipi
150
100
50
0
% cells
0 50 100
PC9
+ Tipi
100
60
40
20
0
0 20 40 60 80
days of treatment
HCC827
+ Tipi
200
150
100
50
0
0 50 100
D
PC9 subclone
+ Osimertinib
> 2 mois
+ Tipifarnib 1 µM
Determination of cell viability
parental PC9
G7
F7
B7
H7
H5
H10
B4
G3
A7
D7
Osimertinib-
fully resistant clones
Tipifarnib sensitivity (%)
100
80
60
40
20
0

Supplementary Figure 13: Response to farnesyltansferase inhibitors in association with targeted therapies
A: Percentage of H23 ($KRAS^{G12C}$), H3122 (ALK^{EML4}) and A375 ($BRAF^{V600E}$) cells treated with their corresponding TT, respectively sotorasib (soto), lorlatinib (lorla) and dabrafenib (dabra) at 1 µM, alone or in combination with tipifarnib at 0.1 µM. Data are mean ± SEM. Representative data from *n*=3 independent biological experiments.
B: Crystal violet staining of parental or clonally-derived PC9, HCC4006 and HCC827 cells treated with different farnesyltransferase inhibitors (FTI), alone or in combination with osimertinib at 1 µM. Tipi: tipifarnib (1 µM); CP: CP-609754 (1 µM); Lona: lonafarnib (1 µM); DTC: Drug-tolerant cells (*i.e.* treated for 7 to 10 days with osimertinib at 1 µM); Relapse: cells treated with osimertinib alone or in combination with FTIs until the emergence of resistant proliferative cells in the osimertinib-treated condition (*i.e.* after 30 to 50 days of treatment, depending on the cell line). Representative images from *n*=3 independent biological experiments.
C: Percentage of HCC4006, PC9 and HCC827 cells treated with 1 µM osimertinib alone (red), or in combination with tipifarnib at 1 µM from day 0 (blue) or after early relapse (*i.e.*, soon after increase of the bulk population in the presence of the drug within the same culture well; green) . Data are mean ± SEM. Representative data from *n*=3 independent biological experiments.
D: PC9 cells were cultured in the presence of 1 µM osimertinib and individual resistant proliferative subclones were grown and amplified in the presence of the drug for at least 2 months. Tipifarnib was then added at 0.1 µM to parental PC9 or 10 different osimertinib-resistant subclones, and tipifarnib sensitivity was determined based on the percentage of surviving cells after 5 days of treatment. Representative data from *n*=3 independent biological experiments.
Source data are provided as a Source data file.

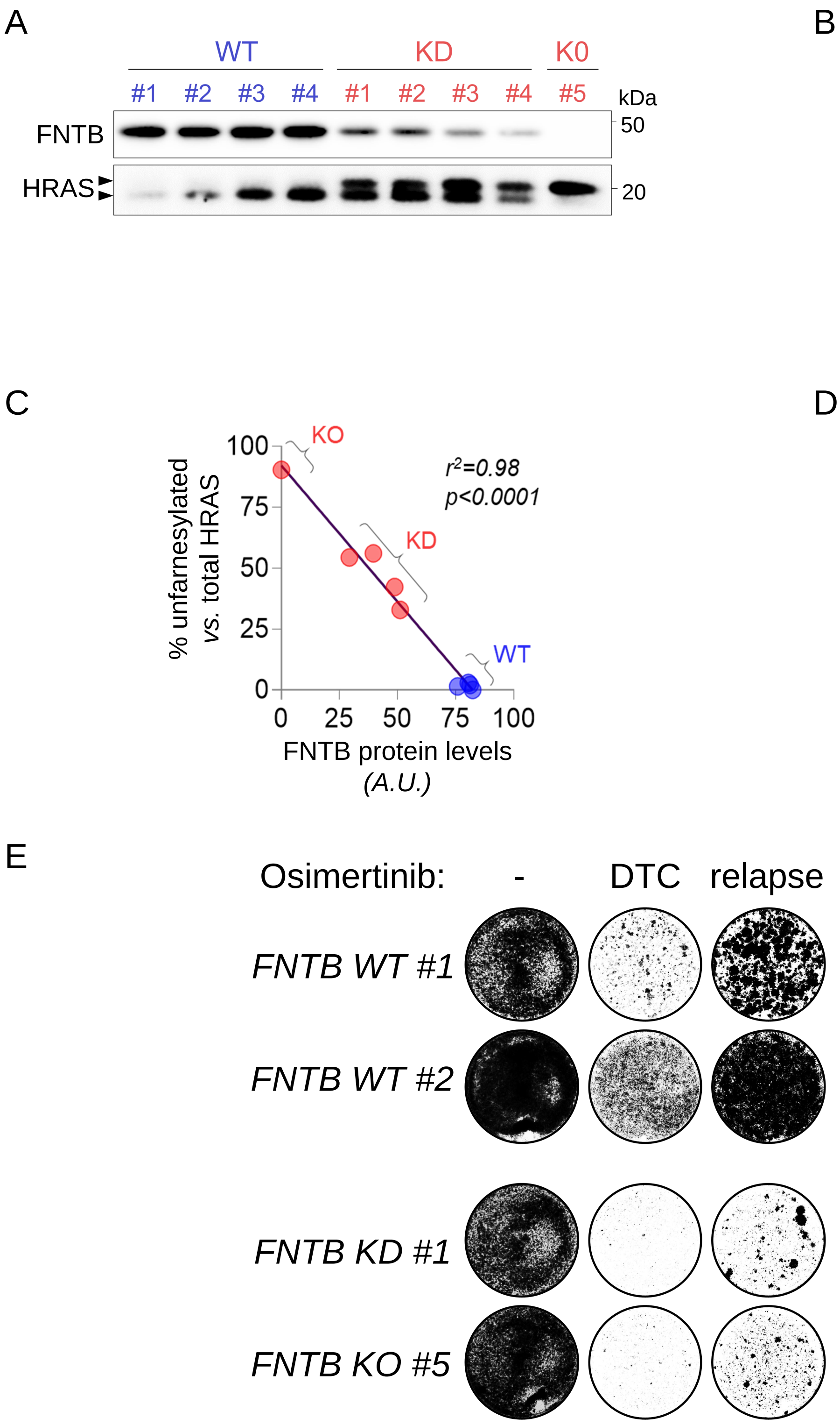

Supplementary Figure 14: Genetic depletion of farnesyltransferase beta subunit

A: Western blot analysis of FNTB level and HRAS farnesylation status in FNTB WT, KD and KO clones. Representative blots from *n*=3 independent biological experiments. Upper arrows show unfarnesylated and lower arrows show farnesylated protein.

B: Quantification of the percentage of unfarnesylated *vs* total HRAS protein in the different clones shown in (A). Representative data from *n*=3 independent biological experiments.

C: Correlation between the percentage of unfarnesylated HRAS and FNTB protein levels, based on (**A**). Representative data from *n*=3 independent biological experiments. *p*-value was calculated using simple linear regression analysis. A.U.: Arbitrary Unit.

D: Characteristic western blot shift of different farnesylated proteins in response to tipifarnib at 0.1 µM. Upper arrows show unfarnesylated and lower arrows show farnesylated protein. Representative blots from *n*=3 independent biological experiments.

E: Crystal violet staining of indicated FNTB WT, KD and KO clones, treated or not with osimertinib 1 µM until DTC (7 to 10 days of treatment) and at relapse (4 weeks of treatment). Representative images from *n*=2 independent biological experiments.

Source data are provided as a Source data file.

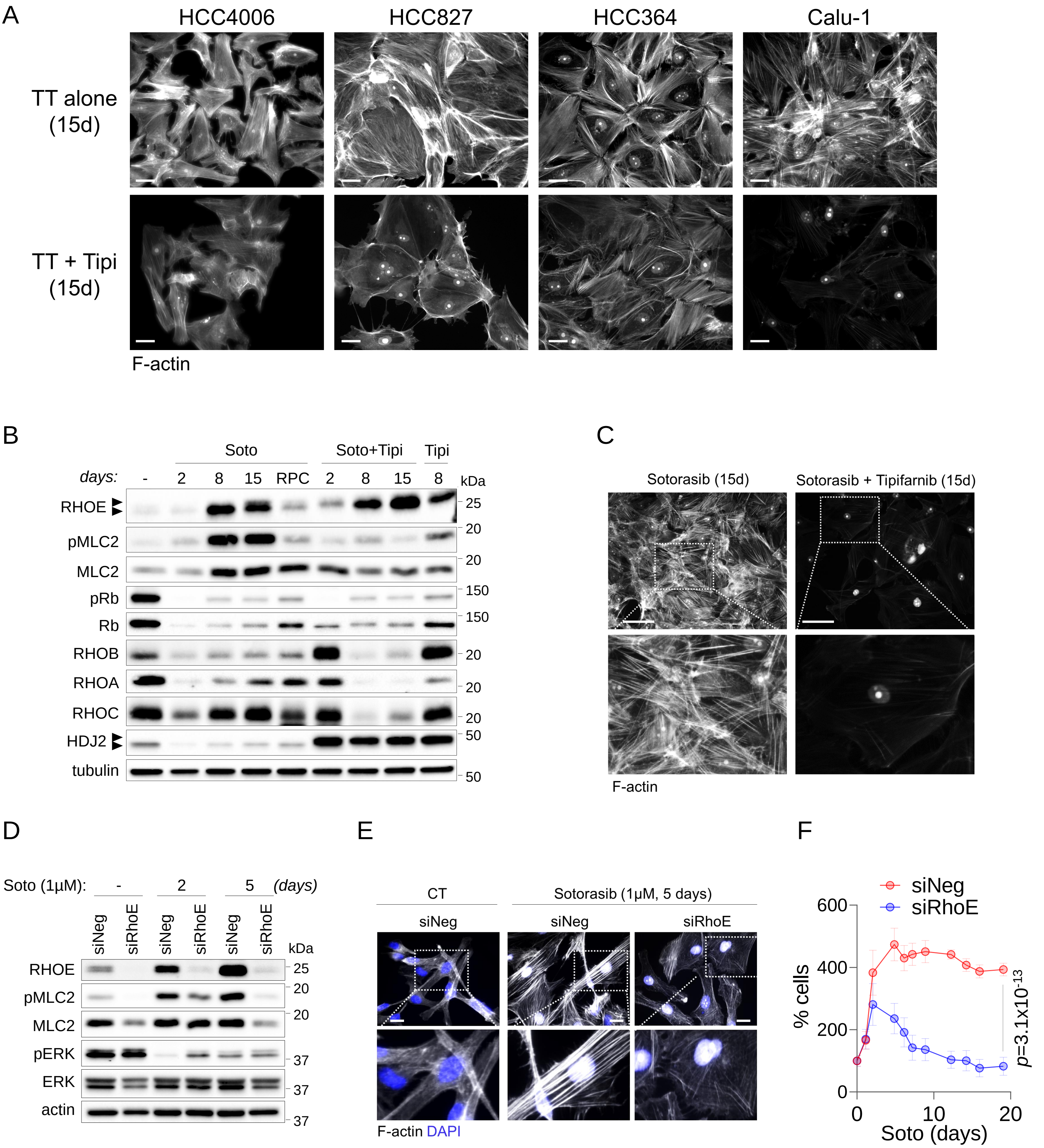
A
HCC4006
HCC827
HCC364
Calu-1
TT alone (15d)
TT + Tipi (15d)
F-actin
B
Soto
Soto+Tipi
Tipi
days:
-
2
8
15
RPC
kDa
RHOE
pMLC2
MLC2
pRb
Rb
RHOB
RHOA
RHOC
HDJ2
tubulin
25
20
150
50
C
Sotorasib (15d)
Sotorasib + Tipifarnib (15d)
D
Soto (1µM):
(days)
siNeg
siRhoE
pERK
ERK
actin
37
E
CT
Sotorasib (1µM, 5 days)
F-actin DAPI
F
siNeg
siRhoE
% cells
Soto (days)
p=3.1x10-13
600
400
200
0
10

Supplementary Figure 15: Farnesylated RHOE mediates the contractile phenotype in response to sotorasib in a KRAS^{G12C} model

A: Phalloidin F-actin staining of HCC4006, HCC827, HCC364 and Calu-1 cells treated for 15 days with their respective targeted therapy at 1 µM (EGFR^{mut}: osimertinib, BRAF^{mut}: dabrafenib, KRAS^{mut}: sotorasib) alone or in combination with 0.1 µM tipifarnib. Scale bar: 20 µm. Representative images from *n*=3 independent biological experiments.

B: Western blot analysis of RhoGTPases and MLC2 pathway in Calu-1 cells treated with 1 µM sotorasib, 0.1 µM tipifarnib or the combination (soto+tipi) at the indicated times. Upper arrows show unfarnesylated and lower arrows show farnesylated protein. Representative blots from *n*=3 independent biological experiments.

C: Phalloidin F-actin staining of Calu-1 cells treated for 15 days with 1 µM sotorasib alone or in combination with 0.1µM tipifarnib. Scale bar: 50 µm. Representative images from *n*=3 independent biological experiments.

D: Western blot analysis of RhoE and total or phospho-MLC2 in Calu-1 cells transfected with siRNA control (siNeg) or targeting RhoE (siRhoE) and treated with 1 µM sotorasib for 5 days. Representative blots from *n*=3 independent biological experiments.

E: Phalloidin F-actin staining of Calu-1 cells transfected with siRNA control (siNeg) or targeting RhoE (siRhoE) and treated with 1 µM sotorasib for 5 days. Scale bar: 20 µm. Representative images from *n*=3 independent biological experiments.

F: Incucyte monitoring of Calu-1 cells transfected with siRNA control (siNeg) or targeting RhoE (siRhoE) and treated with 1 µM sotorasib. Data are mean ± SEM; *p*-value was calculated using two-tailed unpaired *t*-test. Representative blots from *n*=3 independent biological experiments.

Source data are provided as a Source data file.

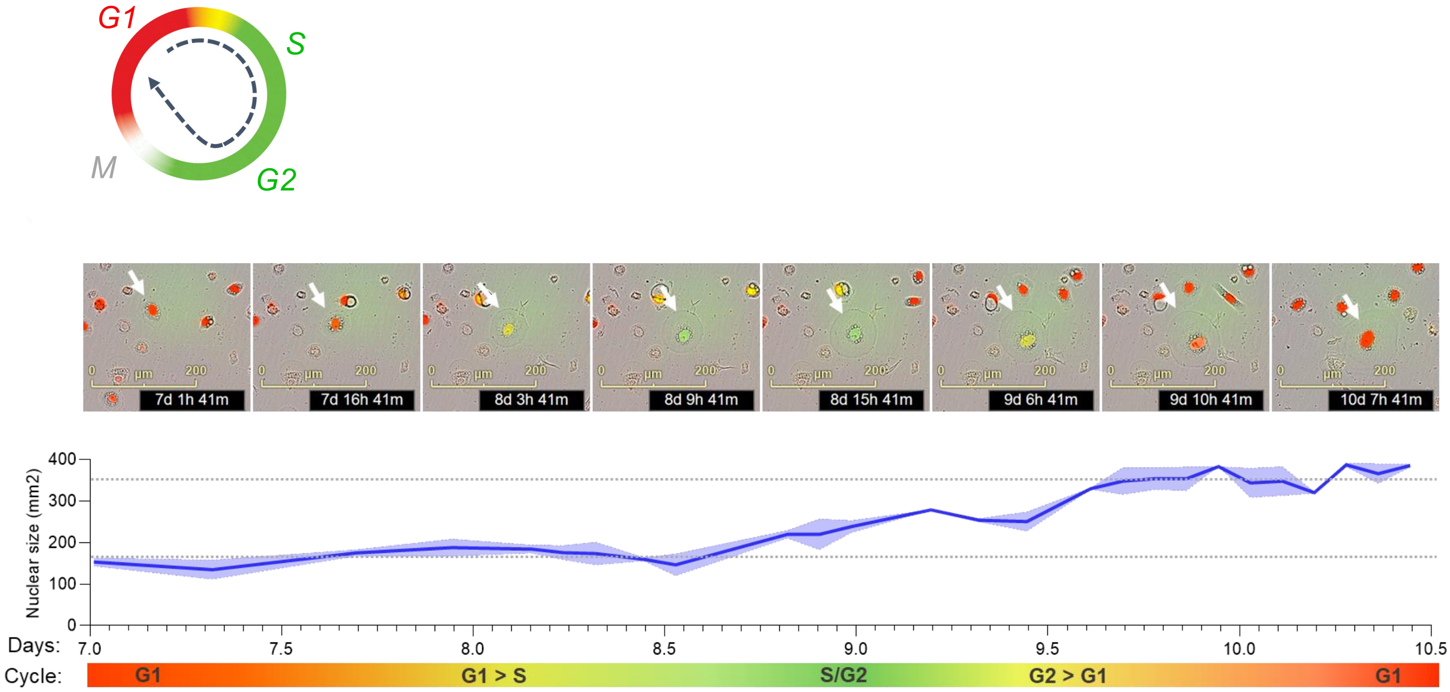

Supplementary Figure 16: Endoreplication process in response to osimertinib in PC9 cells

Top panel: Incucyte monitoring of a PC9 subclonal cell treated with osimertinib (1 µM) showing an endoreplication process. Bottom panel: Quantification of nuclear size of the same PC9 subclonal cell under osimertinib (1 µM) treatment. Data are mean ± SEM. Representative data from *n*=3 independent biological experiments.

Source data are provided as a Source data file.

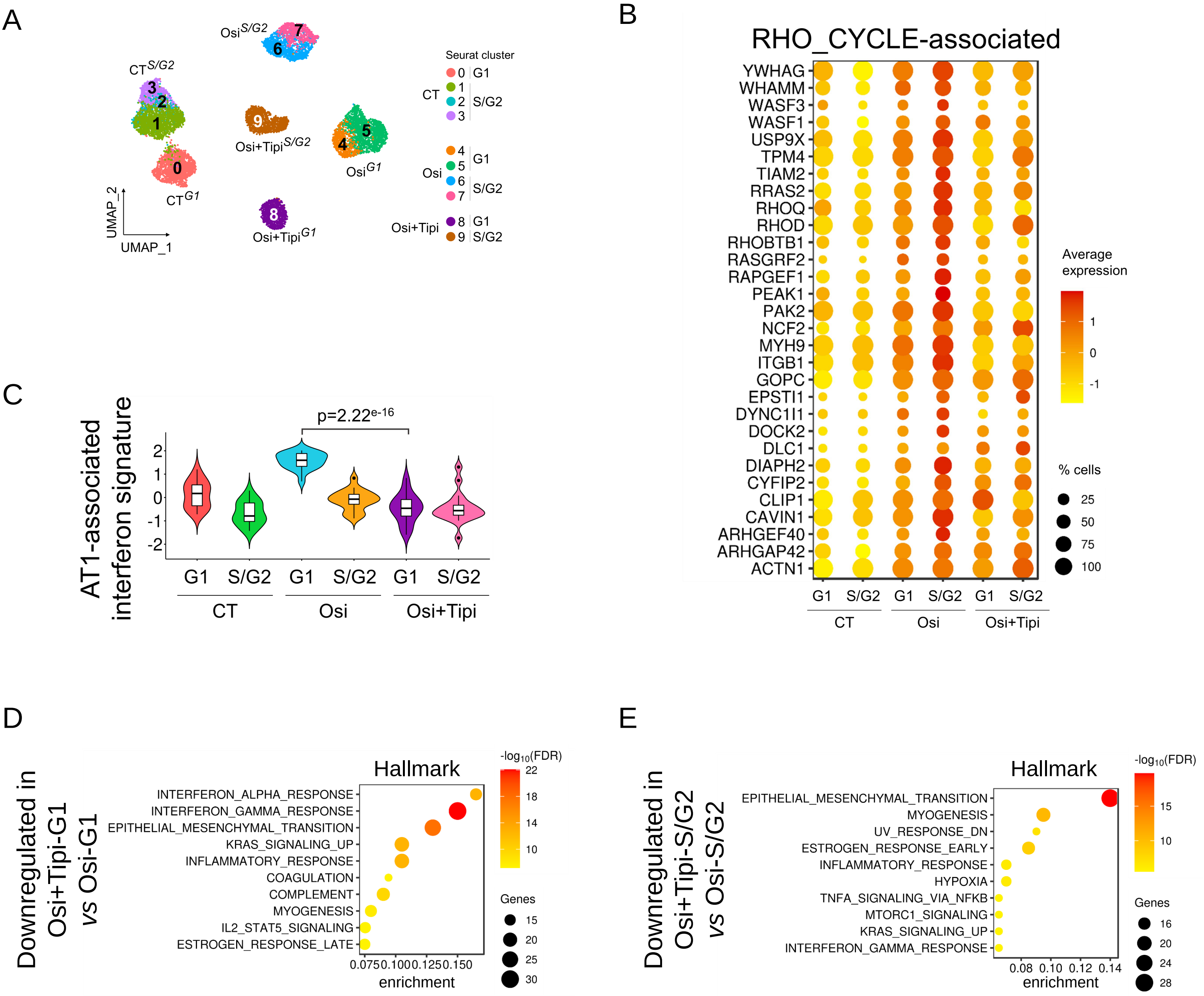

Supplementary Figure 17: Tipifarnib alters osimertinib-induced transcriptomic reprogramming of EGFR-mutant tumor cells

A: UMAP plot of the different clusters from Seurat analysis of untreated, osimertinib-treated and osimertinib+tipifarnib-treated HCC4006 subclonal cells obtained after scRNAseq.

B: Average expression of genes involved in the RHO_CYCLE-associated signature in G1 and S/G2 populations of untreated, osimertinib-treated and osimertinib+tipifarnib-treated cells.

C: Violin plots representing the distribution of z-score values of AT1-associated interferon signature in G1 and S/G2 populations of untreated, osimertinib-treated and osimertinib+tipifarnib-treated cells. The box plots within violin plos display 25th (lower bound), 50th (center, median), and 75th (upper bound) percentiles, with whiskers generated with the Tukey method. CT-G1, n=1678; O-G1, n=2097, OT-G1, n=897; CT-SG2, n=2654; O-SG2, n=1963; OT-SG2, n=1266. *p*-value was calculated using two-sided Wilcoxon test. **D-E**: Dot plot of the top gene signatures downregulated in osimertinib+tipifarnib *vs* osimertinib in the G1 (**D**) or S/G2 (**E**) populations.

Source data are provided as a Source data file.

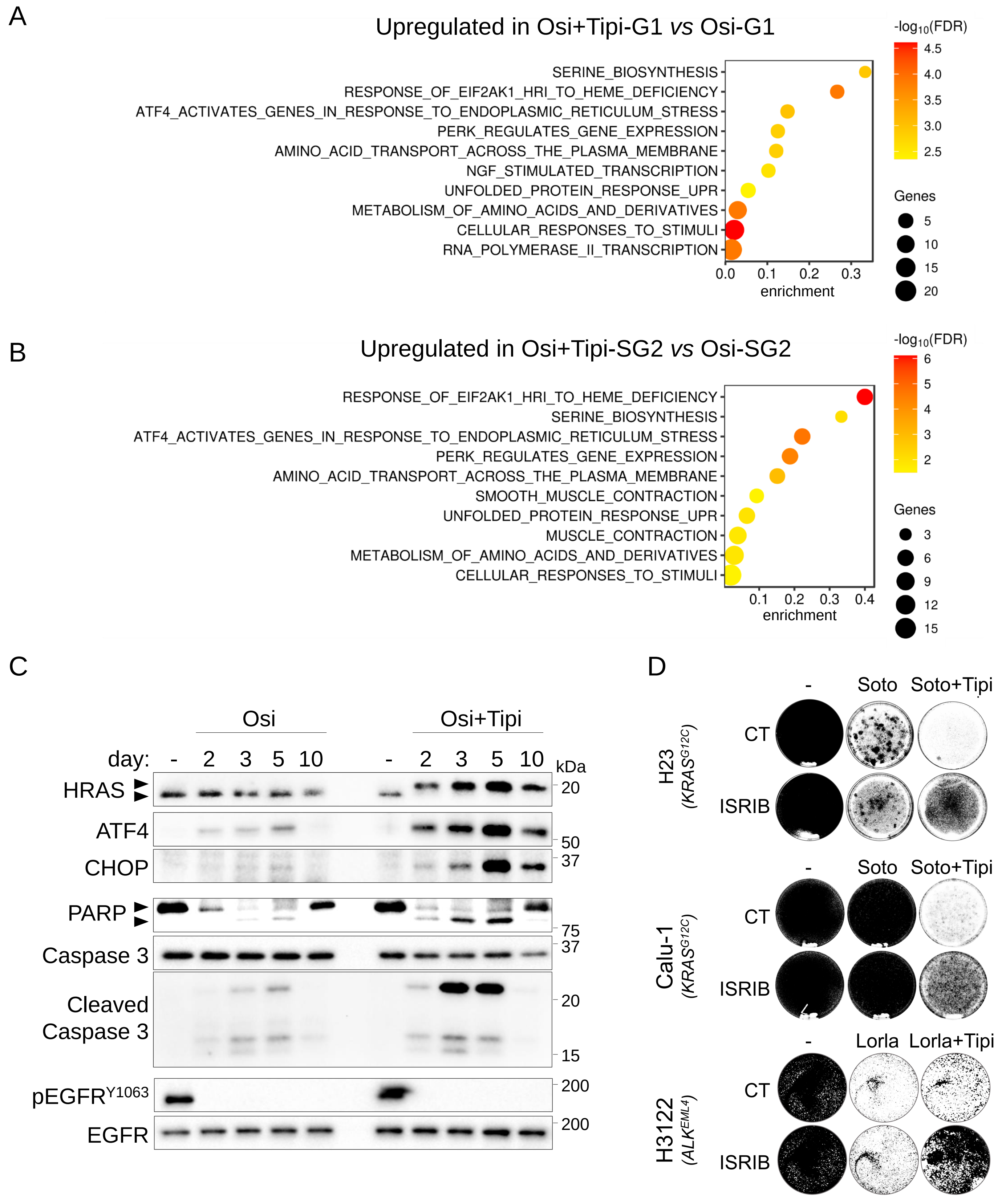

Supplementary Figure 18: Co-treatment with Tipifarnib induces Integrated Stress Response (ISR)-dependent apoptosis

A-B: Dot plot of the top gene signatures upregulated by osimertinib+tipifarnib *vs* osimertinib in the G1 (**A**) or S/G2 (**B**) populations

C: Western blot analysis of proteins related to EGFR pathway (phospho-EGFR, EGFR), farnesyltransferase inhibitor efficacy (HRAS), apoptosis (PARP and caspase-3) and ISR (ATF4, CHOP) on HCC4006 clonal cells treated with osimertinib (1 µM) alone or in combination with tipifarnib (1 µM). For HRAS: upper arrow shows unfarnesylated and lower arrow shows farnesylated protein; for PARP: upper arrow shows total and lower arrow shows cleaved protein. Representative blots from *n*=3 independent biological experiments.

D: Crystal violet staining of H23 (top), Calu-1 (middle) and H3122 (bottom) cells pre-treated or not for 24h with integrated stress response inhibitor (ISRIB, 1 µM) alone or in combination with 1 µM sotorasib (H23 and Calu-1) or lorlatinib (H3122) with or without 1 µM tipifanib. Representative images from *n*=3 independent biological experiments.

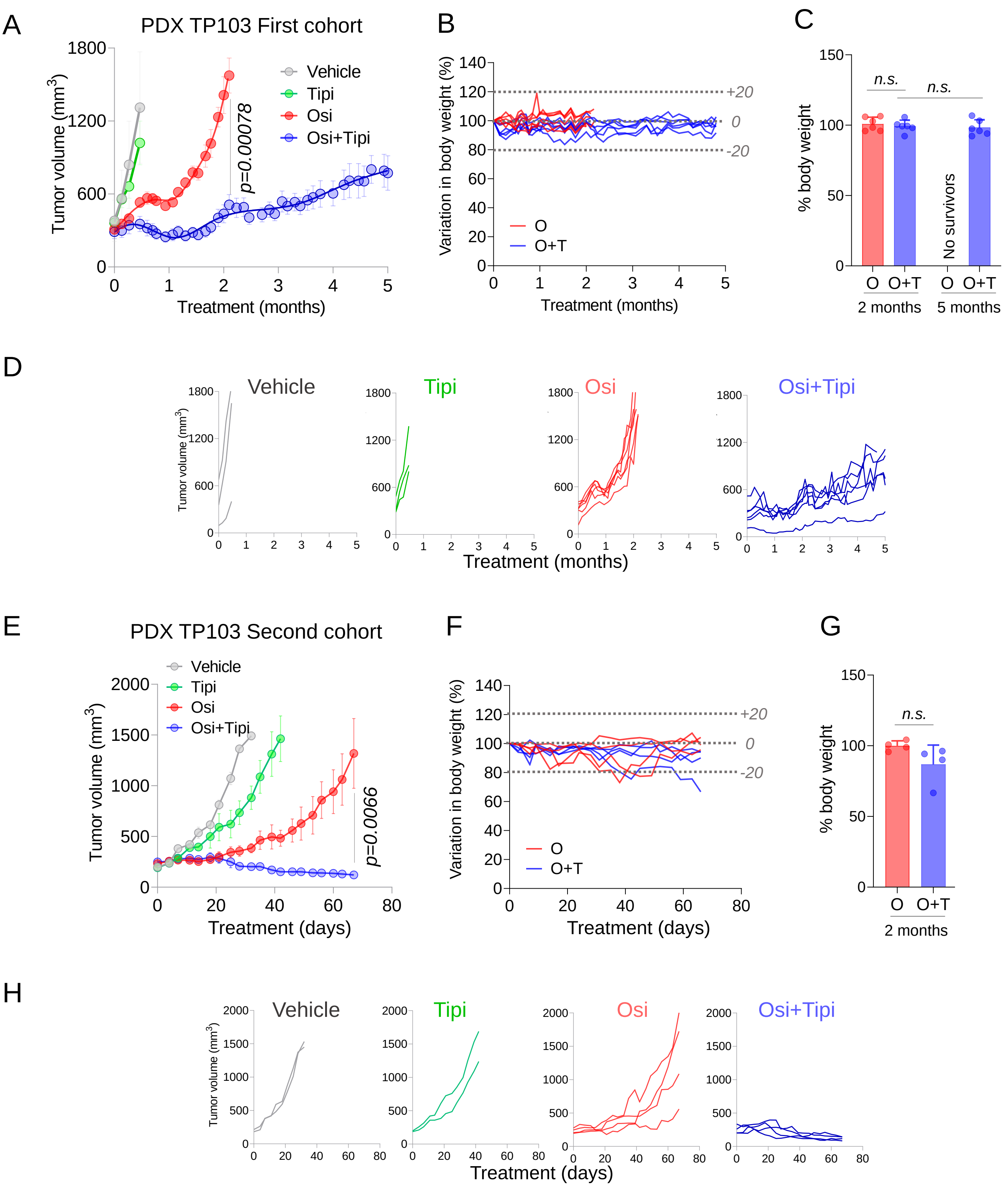

Supplementary Figure 19: Efficacy of tipifarnib in combination with osimertinib in the EGFR-mutant PDX model TP103

A-D: First cohort of mice with PDX TP103. *n*=3 tumors in the vehicle and tipifarnib arms and *n*=6 tumors in the osimertinib and combination arms.

E-H: Second cohort of mice. *n*=2 tumors in the vehicle and tipifarnib arms and *n*=4 tumors in the osimertinib and combination arms.

Mean (**A**, **E**) and individual (**D**, **H**) tumor volume (mm^3) treated 5 days/week with vehicle, tipifarnib (tipi, 80mg/kg, b.i.d.), osimertinib (osi, 5 mg/kg, q.d), or with the combo (osi + tipi).

Variation in body weight (%) during treatment (**B**, **F**) and at the end of treatment (**C**, **G**) with osimertinib and the combination treatments. Data shown in **A**, and **E** are mean ±SEM., data shown in **C**, and **G** are mean ±SD.; n.s.: not significant; *p*-value was calculated using two-tailed unpaired *t*-test.

Source data are provided as a Source data file.

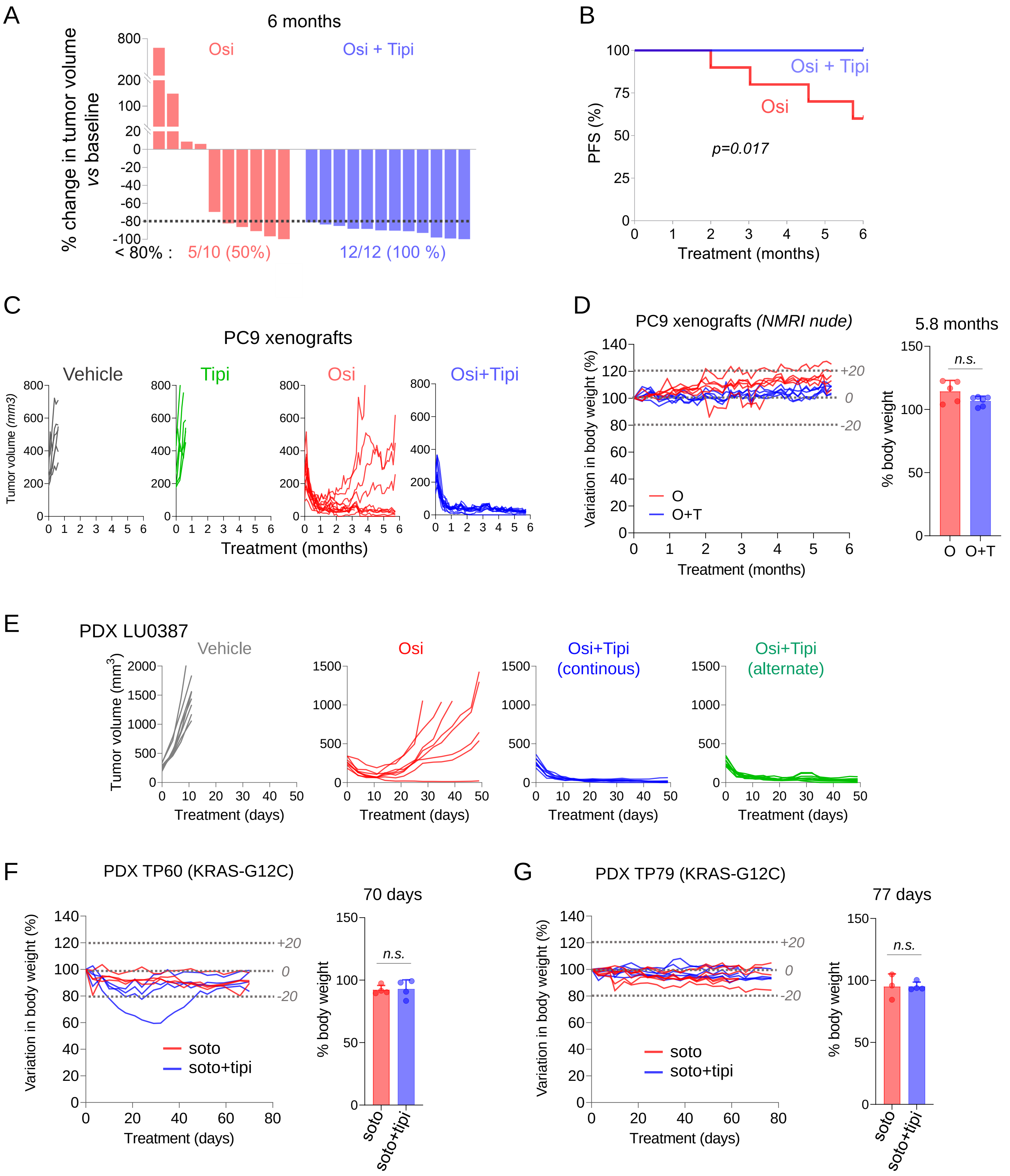
A
6 months
% change in tumor volume vs baseline
Osi
Osi + Tipi
< 80% : 5/10 (50%) 12/12 (100 %)
B
PFS (%)
Osi + Tipi
Osi
p=0.017
Treatment (months)
C
PC9 xenografts
Vehicle
Tipi
Osi
Osi+Tipi
Tumor volume (mm3)
Treatment (months)
D
PC9 xenografts (NMRI nude)
Variation in body weight (%)
+20
0
-20
O
O+T
Treatment (months)
5.8 months
n.s.
% body weight
O
O+T
E
PDX LU0387
Vehicle
Osi
Osi+Tipi (continous)
Osi+Tipi (alternate)
Tumor volume (mm3)
Treatment (days)
F
PDX TP60 (KRAS-G12C)
Variation in body weight (%)
soto
soto+tipi
Treatment (days)
70 days
n.s.
% body weight
soto
soto+tipi
G
PDX TP79 (KRAS-G12C)
Variation in body weight (%)
soto
soto+tipi
Treatment (days)
77 days
n.s.
% body weight
soto
soto+tipi

Supplementary Figure 20: Efficacy of tipifarnib in combination with osimertinib or sotorasib in several EGFR- or KRAS-mutant models

A: Change in tumor volume *vs* baseline of PC9 xenografts after 6 months of treatment with osimertinib or a combination of osimertinib and tipifarnib.

B: Progression-free survival of mice with PC9 xenografts treated with osimertinib or a combination of osimertinib and tipifarnib. *p*-value was calculated using log-rank Mantel-Cox test

C: Individual tumor volume (mm3) of PC9 xenografts treated 5 days/week with vehicle, tipifarnib (tipi, 80mg/kg, b.i.d.), osimertinib (osi, 5 mg/kg, q.d), or by the combo (osi + tipi). *n*=6 tumors in the vehicle and tipifarnib arms, *n*=10 in the osimertinib arm and *n*=12 in the combination arms.

D: Variation of body weight (%) along treatment course (left) and at 5.8 months (right) for the cohort of mice xenografted with PC9 cells. Mean ± SD, n.s.: not significant; two-sided t-tests.

E: Individual tumor volume (mm^3) of PDX model LU0387 treated 5 days/week with vehicle, osimertinib (osi, 25 mg/kg, q.d), or by the combo (osi + tipi 60 mg/kg, continuously b.i.d, or intermittently 1 week on 1 week off).

F: Variation of body weight (%) along treatment course (left) and at 70 days (right) for the cohort of mice with the PDX TP60. Mice were treated with sotorasib (soto, 30 mg/kg, q.d, *n*=5) or by the combo (soto + tipi, *n*=6). Mean ±SD, n.s.= not significant; two-sided t-tests.

G: Variation of body weight (%) along treatment course (left) and at 77 days (right) for the cohort of mice with the PDX TP79. Mice were treated with sotorasib (soto, 30 mg/kg, q.d, *n*=7) or the combo (soto + tipi, *n*=7). Mean ±SD, n.s.= not significant; two-sided t-tests.

Source data are provided as a Source data file.

Supplementary Table 1: Description of the EGFR-mutant models used for the determination of the drug-tolerant signature

Model	type	EGFR mutation	EGFR-TKI	Treatment duration	GEO accession number	Reference
PC9	cell line	ΔE746-A750	Erlotinib, 1 µM	21 days	GSE249721	this study
HCC827	cell line	ΔE749-A750	Osimertinib, 1 µM	11 days	GSE249721	this study
HCC4006	cell line	ΔL747-E749, A750P	Osimertinib, 1 µM	20 days	GSE248450	this study
H3255	cell line	L858R	Erlotinib, 1 µM	21 days	GSE249721	this study
H1975	cell line	L858R, T790M	Osimertinib, 1 µM	21 days	GSE193259	Criscione *et al.*[3]
HCC2935	cell line	ΔE746-T751, S752I	Osimertinib, 1 µM	21 days	GSE193259	Criscione *et al.*[3]
PHLC137	PDX	Δ746-A750	Erlotinib, 50 mg/kg	30 days	GSE198672	Moghal *et al.*[2]

Supplementary Table 2: Reactome analysis of cytoskeletal-related genes (class ID: PC00085) upregulated in healthy lungs vs LUAD.

Cytoskeletal-related genes (class ID: PC00085) upregulated in healthy lungs *vs* lung adenocarcinoma (LUAD) were analyzed using Reactome pathway analyzer (https://reactome.org).

Pathway name	Entities				Reactions	
	found	ratio	p-value	FDR	found	ratio
Striated Muscle Contraction	8/40	0.003	2.63E-10	4.79E-08	4/4	2.95E-04
Muscle contraction	12/213	0.015	1.29E-08	1.18E-06	10/42	0.003
Intraflagellar transport	7/56	0.004	8.69E-08	5.22E-06	8/12	8.84E-04
Kinesins	6/68	0.005	5.52E-06	2.48E-04	2/14	0.001
Golgi-to-ER retrograde transport	7/148	0.01	4.88E-05	0.002	4/18	0.001
Cilium Assembly	8/210	0.015	6.31E-05	0.002	18/50	0.004
COPI-dependant Golgi-to-ER retrograde traffic	6/107	0.007	6.87E-05	0.002	2/11	8.10E-04
Aggrephagy	4/47	0.003	2.50E-04	0.005	5/15	0.001
Smooth Muscle Contraction	4/49	0.003	2.93E-04	0.005	5/11	8.10E-04
Assembly and cell surface presentation of NMDA receptors	4/49	0.003	2.93E-04	0.005	2/23	0.002
Microtubule-dependant trafficking of connexons from Golgi to the plasma membrane	3/22	0.002	4.02E-04	0.006	1/2	1.47E-04
Macroautophagy	6/150	0.011	4.19E-04	0.006	17/87	0.006
Carboxyterminal post-translational modifications of tubulin	4/55	0.004	4.51E-04	0.006	6/6	4.42E-04
Transport of connexons to the plasma membrane	3/23	0.002	4.57E-04	0.006	1/3	2.21E-04
Intra-Golgi and retrograde Golgi-to-ER traffic	7/219	0.015	5.27E-04	0.006	4/48	0.004
Post-chaperonin tubulin folding pathway	3/25	0.002	5.82E-04	0.006	9/9	6.63E-04
Autophagy	6/166	0.012	7.10E-04	0.007	17/108	0.008
COPI-independant Golgi-to-ER retrograde traffic	4/63	0.004	7.48E-04	0.007	2/7	5.16E-04
Formation of tubulin folding intermediates by CCT/TriC	3/30	0.002	9.83E-04	0.009	2/2	1.47E-04

Supplementary Table 3: List of antibodies with providers, references and dilutions, used for Western Blot experiments

Target	Provider	Reference	Lot number	Dilution
p-ERK1/2 Thr202/Tyr204	Cell Signaling	#4370	28	1/1000
ERK	Santa Cruz Biotechnology	Sc-93	H2614	1/2000
p-EGFR Tyr1068	Cell Signaling	#2234	22	1/2000
EGFR	Cell Signaling	#4267	24	1/2000
N-cadherin	Cell Signaling	#4061	3	1/500
p-RB Ser807/811	Cell Signaling	#8516	8	1/1000
RB	Cell Signaling	#9309	14	1/2000
p27	Cell Signaling	#3686	8	1/1000
p53	Cell Signaling	#48818	1	1/1000
Cyclin D1	Cell Signaling	#2978	13	1/1000
Cyclin E1	Cell Signaling	#20808	3	1/1000
p-MLC2 Ser19	Cell Signaling	#3671	7	1/1000
MLC2	Cell Signaling	#8505	6	1/2000
PARP	Cell Signaling	#9542	15	1/1000
Caspase-3 cleaved	Cell Signaling	#9661	47	1/1000
Caspase-3	Cell Signaling	#9662	19	1/1000
HRAS	Santa Cruz Biotechnology	sc520	K207	1/1000
FNTB	Abclonal	A2611	4000001923	1/2000
ATF4	Cell Signaling	#11815	6	1/1000
CHOP	Cell Signaling	#2895	14	1/1000
AGER	Cell Signaling	#55222	1	1/1000
RhoA	Santa Cruz Biotechnology	Sc-418	J0914	1/1000
RhoB	ProteinTech	14326-1AP	102742	1/1000
RhoC	Cell Signaling	#3430	5	1/5000
RhoE	Cell Signaling	#3664	1	1/1000
LaminB1	Abcam	ab16048	1022148-1	1/2000
HDJ2	Abcam	ab126774	GR82996-12	1/1000
Actin	Merck Millipore	MAB1501	3845682	1/50000
Tubulin	Sigma	T5168	84283	1/50000

Supplementary Table 4: List of inhibitors, references and concentration, used for *in vitro* experiments

Target	Drug	Provider	Reference	Concentrations used for in vitro experiments
EGFR	Erlotinib (OSI-744, CP-358774, NSC 718781)	LC Laboratories	E4997	1 µM
	Osimertinib (AZD9291)	LC Laboratories	0-7200	1 µM
KRAS G12C	Sotorasib	TargetMol	T8684	1 µM
ALK-EML4	Lorlatinib	TargetMol	T3061	1 µM
BRAF V600E	Dabrafenib	MedChem	HY-14660A	1 µM
Farnesyltransferase	Tipifarnib (R115777, IND 58359)	Kura Oncology		0.1 – 1 µM
	Lonafarnib	Selleckchem	S2797	0.1 – 1 µM
	CP-609754	Kura Oncology		0.1 – 1 µM
	FTI-2153	Kindly provided by Saïd Sebti and Andrew D. Hamilton		1 µM
Géranylgeranyl-transferase I	GGTI-298	Selleckchem	S7466	1 µM
	GGTI-2166	Kindly provided by Saïd Sebti and Andrew D. Hamilton		1 µM
ROCK	Y27632	Selleckchem	S1049	10 µM
	GSK269962A	Selleckchem	S7687	5 µM
RHOA, B, C	TAT-C3	C3 exoenzyme coupled to permeant peptide TAT (1 or 10 µg/ml) were produced and purified in our laboratory using an Akta purifier (GE Healthcare) as previously described (réf: Sahai, E. & Olson, M. F. Purification of TAT-C3 exoenzyme. Meth. Enzymol. 406, 128–140 (2006)).		5-10 µg/ml
Actin	Latrunculin B	Sigma-Aldrich	428020	0.3 µM
Integrated Stress Response	ISRIB	Sigma-Aldrich	SML0843	1 µM

Supplementary Table 5: List of PDX models used for *in vivo* experiments, including frequency, posology and vehicles

Model	Drug	Dose	Frequency	Vehicle
TP103, PC9	Osimertinib	5 mg/kg	Once a day	0,5% carboxymethylcellulose (Sigma, M0512) + 0,1% Tween80 (Sigma, P8074)
TP60, TP79	Sotorasib	30 mg/kg	Once a day	2-hydroxypropryl-cyclodextrin 20% (Sigma, H107)
TP103, TP60, TP79, PC9	Tipifarnib	80 mg/kg	Twice a day	2-hydroxypropryl-cyclodextrin 20% (Sigma, H107)
LU0387	Osimertinib	25 mg/kg	Once a day	5%DMSO (0.24ml), 40% PEG300 (1.92ml), 5% Tween 80
LU0387	Tipifarnib	60 mg/kg	Twice a day	2-hydroxypropryl-cyclodextrin 20% (Sigma, H107

Supplementary references

1. Tirosh, I. *et al.* Single-cell RNA-seq supports a developmental hierarchy in human oligodendroglioma. *Nature* **539**, 309-313 (2016). https://doi.org:10.1038/nature20123
2. Moghal, N. *et al.* Single-Cell Analysis Reveals Transcriptomic Features of Drug-Tolerant Persisters and Stromal Adaptation in a Patient-Derived EGFR-Mutated Lung Adenocarcinoma Xenograft Model. *Journal of thoracic oncology : official publication of the International Association for the Study of Lung Cancer* **18**, 499-515 (2023). https://doi.org:10.1016/j.jtho.2022.12.003
3. Criscione, S. W. *et al.* The landscape of therapeutic vulnerabilities in EGFR inhibitor osimertinib drug tolerant persister cells. *NPJ Precis Oncol* **6**, 95 (2022). https://doi.org:10.1038/s41698-022-00337-w
4. Tanimura, K. *et al.* HER3 activation contributes toward the emergence of ALK inhibitor-tolerant cells in ALK-rearranged lung cancer with mesenchymal features. *NPJ Precis Oncol* **6**, 5 (2022). https://doi.org:10.1038/s41698-021-00250-8
5. Liu, Y. *et al.* Enhancing the Therapeutic Efficacy of KRAS(G12C) Inhibitors in Lung Adenocarcinoma Cell Models by Cotargeting the MAPK Pathway or HSP90. *J Oncol* **2021**, 2721466 (2021). https://doi.org:10.1155/2021/2721466
6. Lin, L. *et al.* The Hippo effector YAP promotes resistance to RAF- and MEK-targeted cancer therapies. *Nat Genet* **47**, 250-256 (2015). https://doi.org:10.1038/ng.3218

Uncropped scans of blots presented in Supplementary Figures

Supp Fig. 2B (1/3)

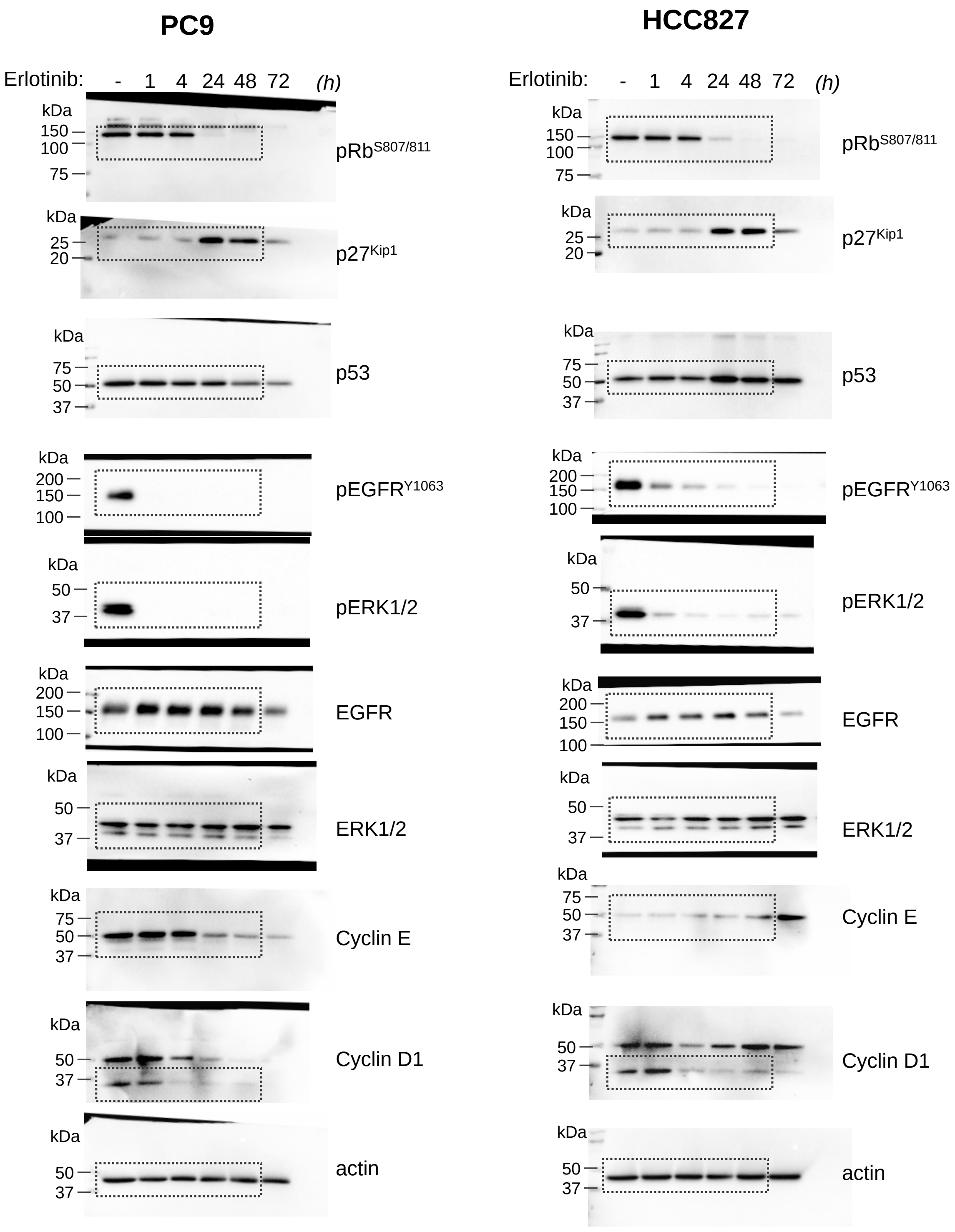

Supp Fig. 2B (2/3)

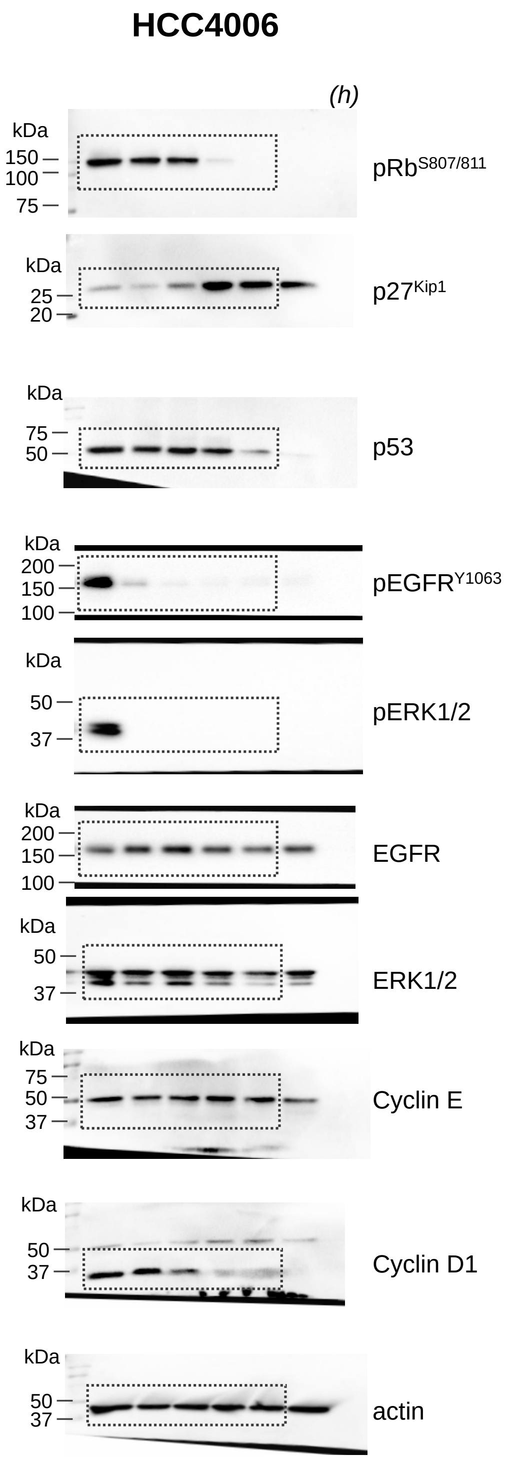

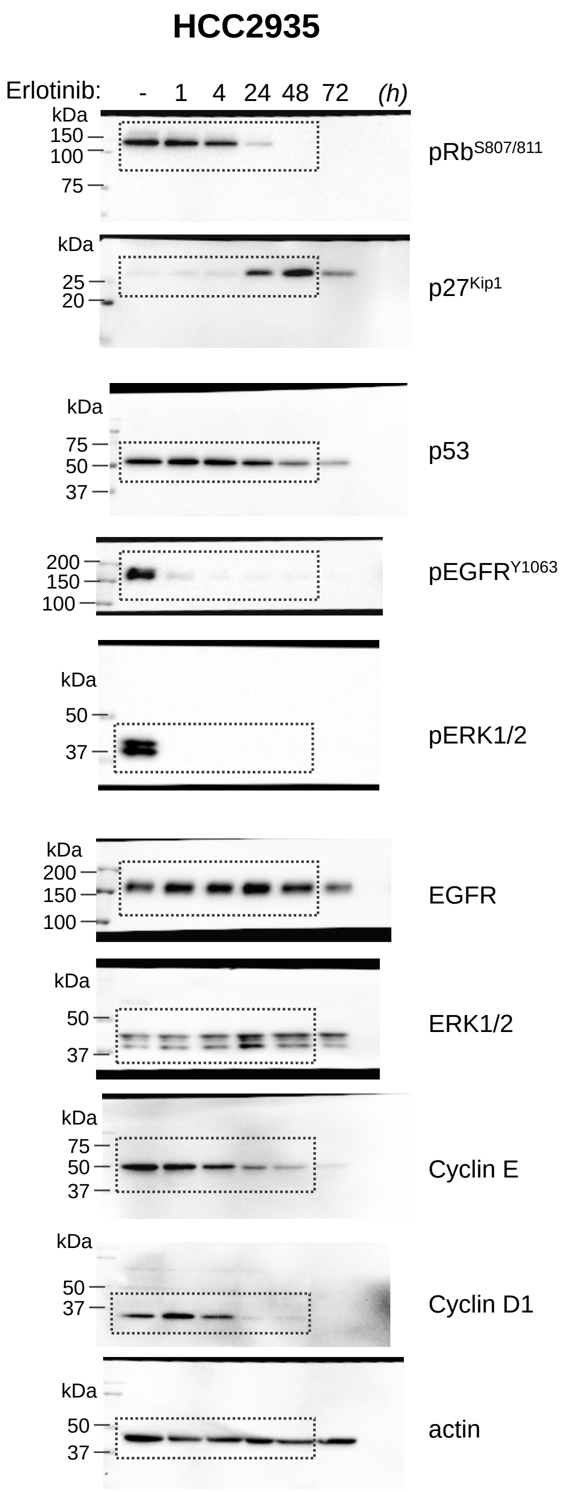

Supp Fig. 2B (3/3)

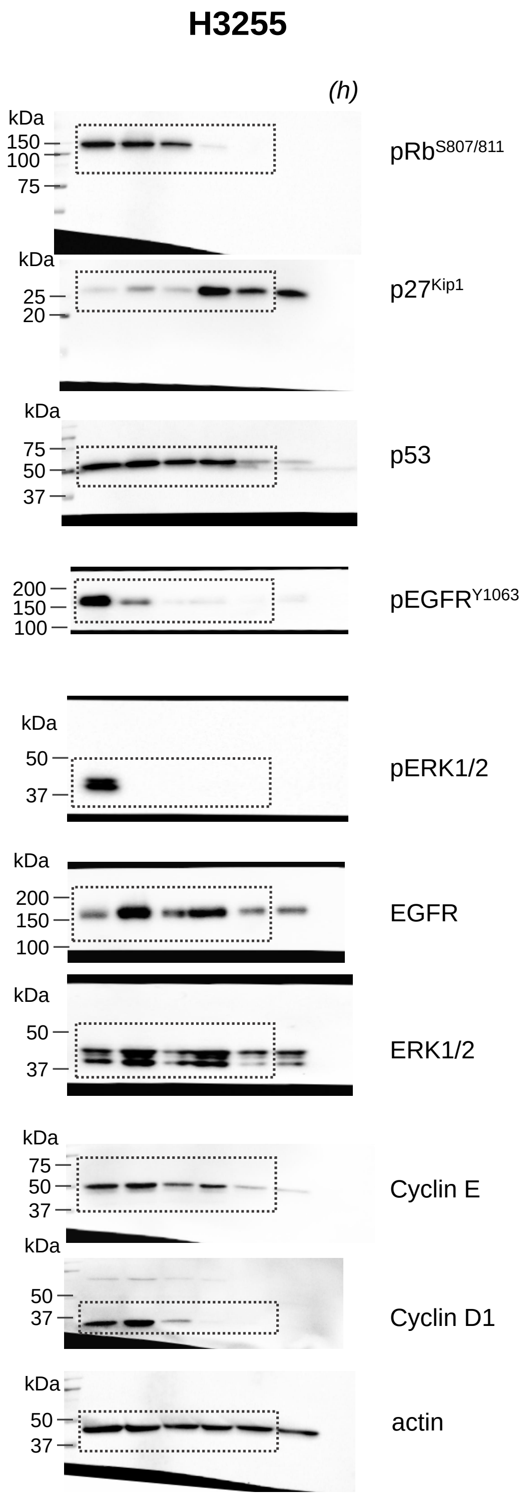

Supp Fig. 3

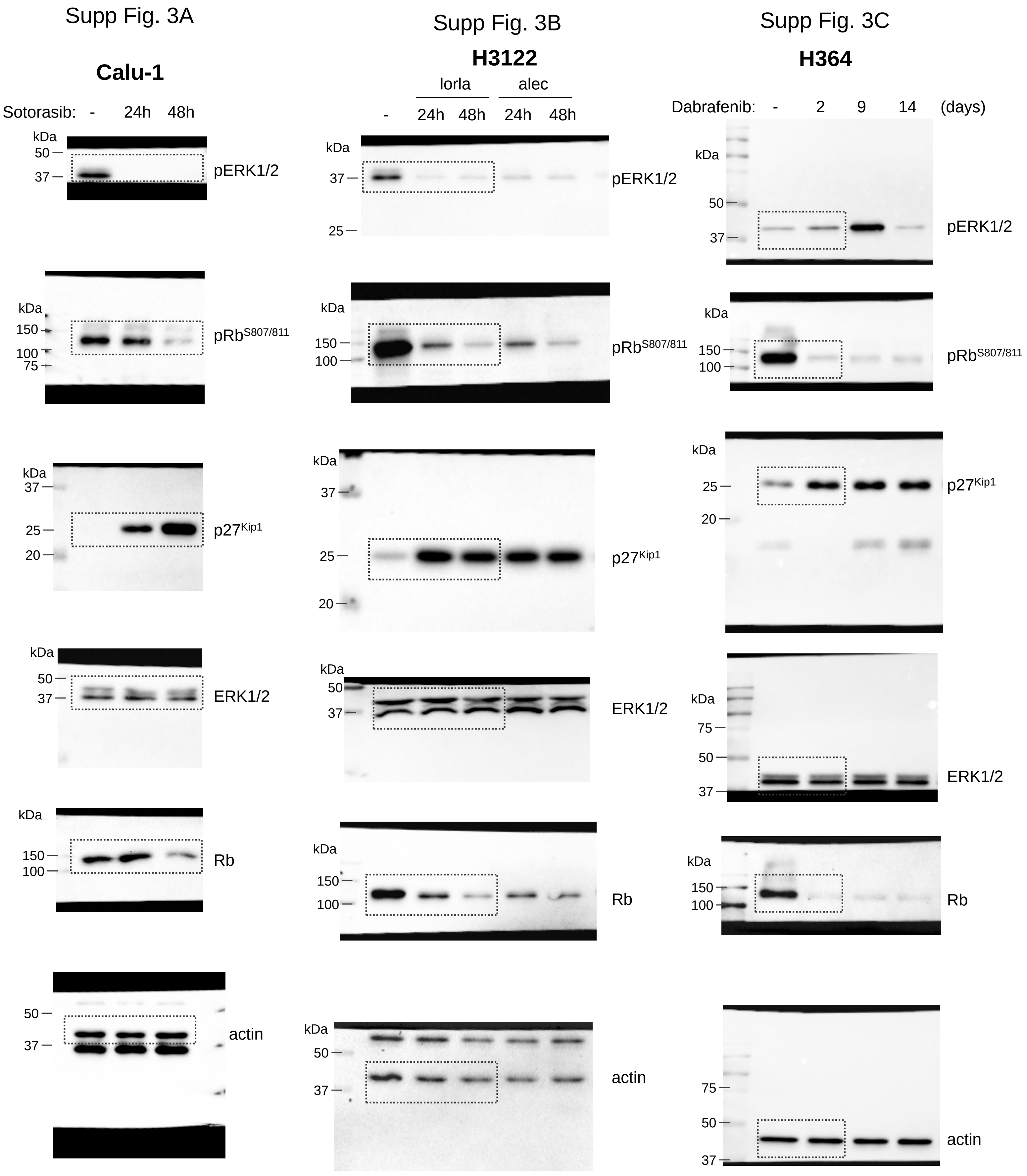

Supp Fig. 3A
Calu-1
Sotorasib: - 24h 48h
kDa
50
37
pERK1/2
150
100
75
pRbS807/811
25
20
p27Kip1
ERK1/2
Rb
actin
Supp Fig. 3B
H3122
lorla alec
- 24h 48h 24h 48h
Supp Fig. 3C
H364
Dabrafenib: - 2 9 14 (days)

Supp Fig. 4H

Calu-1

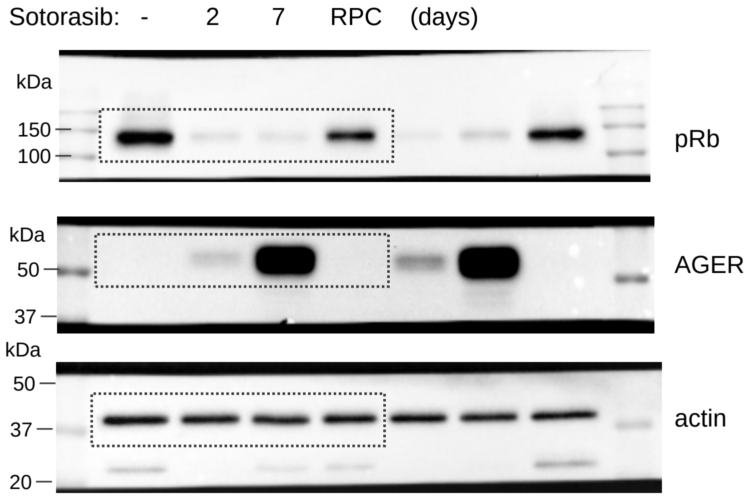

H364

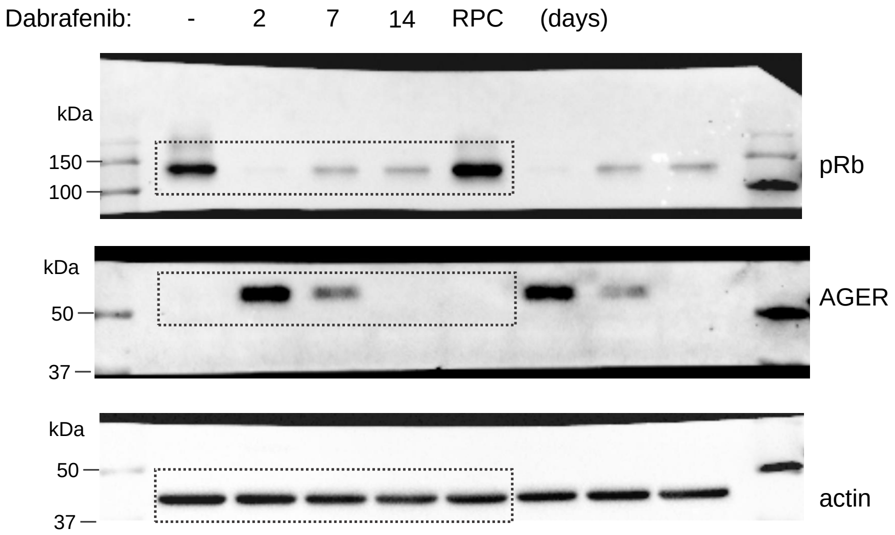

H3122

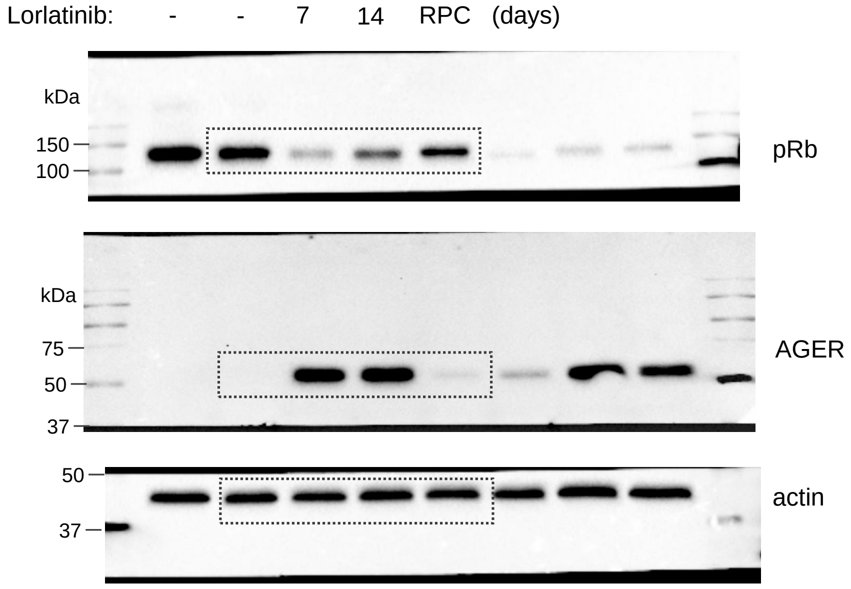

Supp Fig. 11C (1/3)

HCC4006

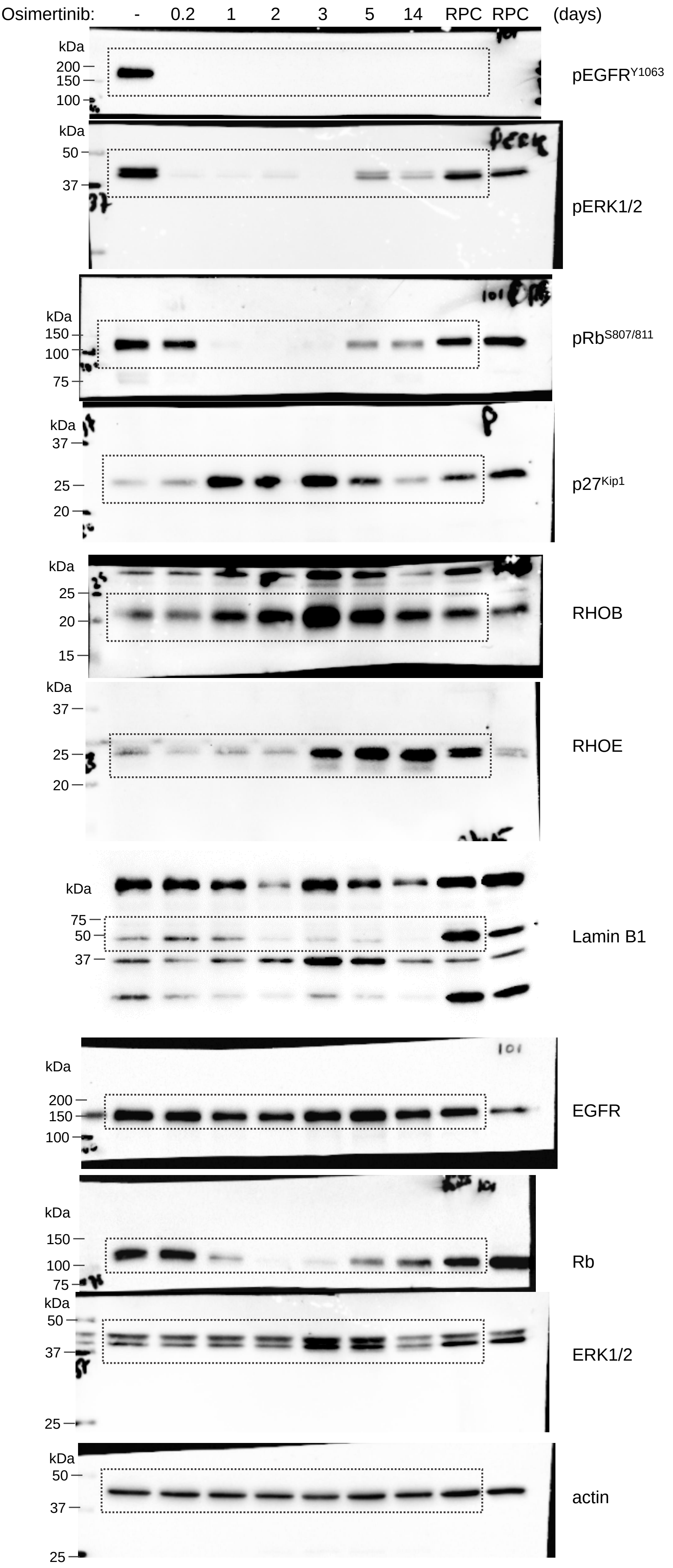

Supp Fig. 11C

(2/3)

PC9

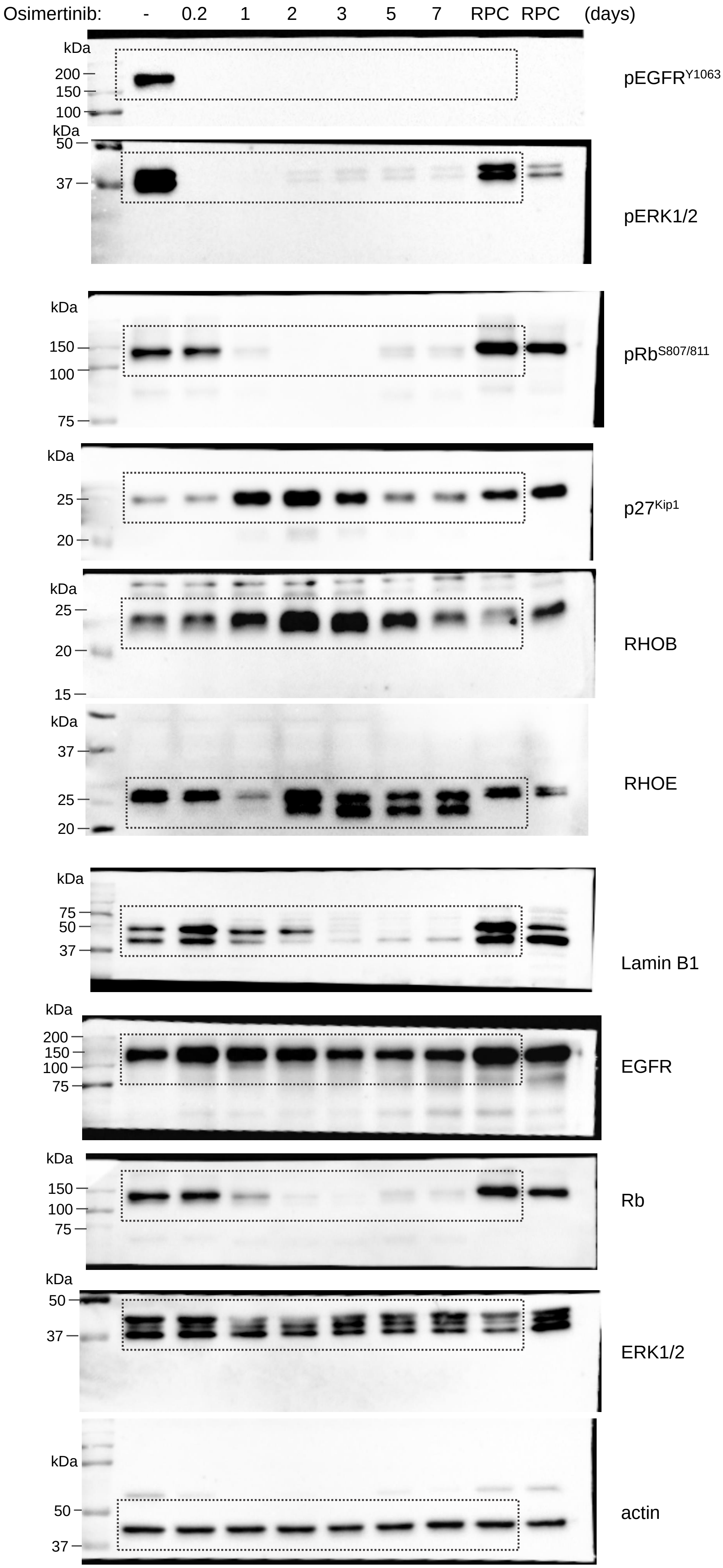

Supp Fig. 11C

(3/3)

HCC827

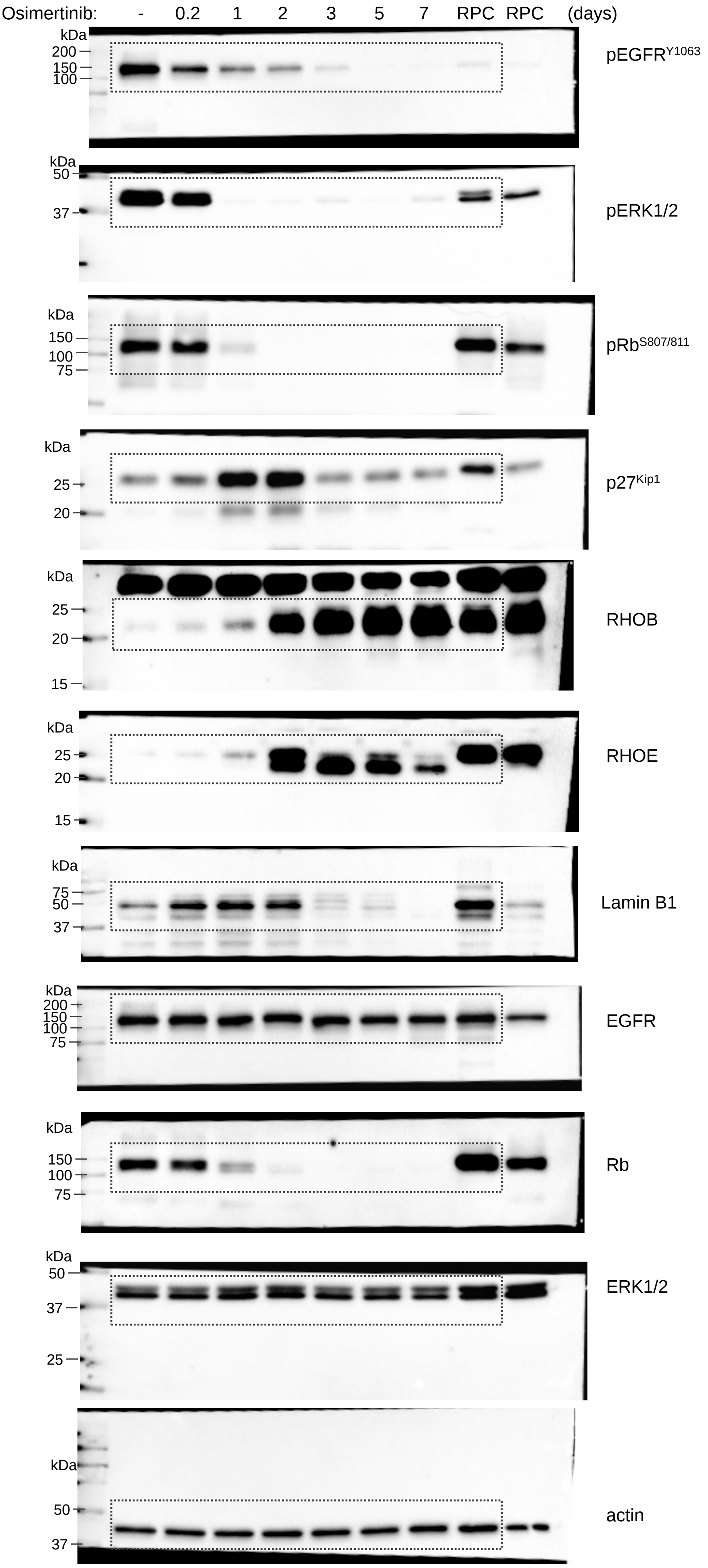

Supplement: Supplementary file 1 — Supplementary information [file 41467_2024_49360_MOESM1_ESM.pdf]
