# Supplementary material for: Farnesyltransferase inhibition overcomes oncogene-addicted non-small cell lung cancer adaptive resistance to targeted therapies
Source: Nat Commun. 2024 Jun 27;15:5345. doi: 10.1038/s41467-024-49360-4 (PMC11211478; doi:10.1038/s41467-024-49360-4)
Supplement: Supplementary file 2 — Reporting Summary [file 41467_2024_49360_MOESM2_ESM.pdf]

nature portfolio

Corresponding author(s): Olivier Calvayrac & Gilles Favre

Last updated by author(s): May 16, 2024

Reporting Summary

Nature Portfolio wishes to improve the reproducibility of the work that we publish. This form provides structure for consistency and transparency in reporting. For further information on Nature Portfolio policies, see our Editorial Policies and the Editorial Policy Checklist.

Statistics

For all statistical analyses, confirm that the following items are present in the figure legend, table legend, main text, or Methods section.

n/a	Confirmed	
☐	☒	The exact sample size (*n*) for each experimental group/condition, given as a discrete number and unit of measurement
☐	☒	A statement on whether measurements were taken from distinct samples or whether the same sample was measured repeatedly
☐	☒	The statistical test(s) used AND whether they are one- or two-sided<br>*Only common tests should be described solely by name; describe more complex techniques in the Methods section.*
☐	☒	A description of all covariates tested
☐	☒	A description of any assumptions or corrections, such as tests of normality and adjustment for multiple comparisons
☐	☒	A full description of the statistical parameters including central tendency (e.g. means) or other basic estimates (e.g. regression coefficient) AND variation (e.g. standard deviation) or associated estimates of uncertainty (e.g. confidence intervals)
☐	☒	For null hypothesis testing, the test statistic (e.g. *F*, *t*, *r*) with confidence intervals, effect sizes, degrees of freedom and *P* value noted<br>*Give P values as exact values whenever suitable.*
☒	☐	For Bayesian analysis, information on the choice of priors and Markov chain Monte Carlo settings
☒	☐	For hierarchical and complex designs, identification of the appropriate level for tests and full reporting of outcomes
☐	☒	Estimates of effect sizes (e.g. Cohen's *d*, Pearson's *r*), indicating how they were calculated

Our web collection on statistics for biologists contains articles on many of the points above.

Software and code

Policy information about availability of computer code

Data collection	All sequencing experiments were performed using a NextSeq 550 (Illumina). For RNAseq experiments, sequences were aligned with RSEM v1.3.3., and for scRNAseq experiments, libraries were generated using the 10X Genomics Chromium Single Cell 3' Kit and sequences were aligned using Cell Ranger 6.0.0.<br>FUCCI-labelled cells were monitored using an IncuCyte S3 (Sartorius). For scRNAseq experiments, red (G1) and green (S/G2) cells were sorted using FACS MELODY (BD Biosciences) using the BD FACSChorus v1.3.3 software.<br>Phase contrast and fluorescence images were acquired using Nikon Eclipse Ti and Zeiss Axio Vert.A1 microscopes.<br>Immunoblots and crystal violet staining were revealed using BioRad Chemidoc MP imaging system.<br>IHC images were retrieved using the NIS-Elements Viewer 5.21 software

Data analysis	RNAseq data were analyzed using DSeq2 v1.34.0. and scRNAseq were analyzed using Seurat v4.0. Other softwares used in this study for scRNAseq analysis were: scvelo v0.2.4, scanpy v1.7.2, anndata v0.7.6, loompy v2.0.16, SingleCellExperiment v1.12.0, scran v1.18.5, and edgeR v3.32.1.<br>All software used are open source, Seurat and Deseq2 libraries are respectively available via CRAN (https://cran.r-project.org/) and Bioconductor (https://bioconductor.org/). Also in R environment, Dynverse packages are available via devtools as "dynverse/dyno". In addition, both scVelo (based on python environmen) and scSignatureExplorer are available via at GitHub at https://github.com/theislab/scvelo and https://github.com/FredPont/spatial, respectively.<br>Incucyte data were analyzed by Incucyte 2020B software.<br>Images were analyzed using Image J 1.52p software<br>GraphPad Prism v9 was used for statistical analysis<br>GSEA analysis were performed using GSEA v4.1.0 software

For manuscripts utilizing custom algorithms or software that are central to the research but not yet described in published literature, software must be made available to editors and reviewers. We strongly encourage code deposition in a community repository (e.g. GitHub). See the Nature Portfolio guidelines for submitting code & software for further information.

Data

Policy information about availability of data

All manuscripts must include a data availability statement. This statement should provide the following information, where applicable:

- Accession codes, unique identifiers, or web links for publicly available datasets
- A description of any restrictions on data availability
- For clinical datasets or third party data, please ensure that the statement adheres to our policy

The RNAseq and scRNAseq data generated in this study are publicly available at the NCBI Gene Expression Omnibus (GEO) database under accession codes GSE249721 and GSE248450, respectively. Source data are provided with this paper. Other publicly available RNA-seq and scRNAseq transcriptomic data used in this study are available through the NCBI GEO database under accession codes GSE198672, GSE193259, GSE164326, GSE188406, and GSE64550. TCGA expression data of human healthy lungs and lung adenocarcinoma used in this study are available at http://firebrowse.org. Source data are provided with this paper.The remaining data are available within the article, supplementary information or source data file. Source data are provided with this paper. Source data are provided as a Source Data file.

Research involving human participants, their data, or biological material

Policy information about studies with human participants or human data. See also policy information about sex, gender (identity/presentation), and sexual orientation and race, ethnicity and racism.

Reporting on sex and gender	*Use the terms sex (biological attribute) and gender (shaped by social and cultural circumstances) carefully in order to avoid confusing both terms. Indicate if findings apply to only one sex or gender; describe whether sex and gender were considered in study design; whether sex and/or gender was determined based on self-reporting or assigned and methods used.*<br>*Provide in the source data disaggregated sex and gender data, where this information has been collected, and if consent has been obtained for sharing of individual-level data; provide overall numbers in this Reporting Summary. Please state if this information has not been collected.*<br>*Report sex- and gender-based analyses where performed, justify reasons for lack of sex- and gender-based analysis.*
Reporting on race, ethnicity, or other socially relevant groupings	*Please specify the socially constructed or socially relevant categorization variable(s) used in your manuscript and explain why they were used. Please note that such variables should not be used as proxies for other socially constructed/relevant variables (for example, race or ethnicity should not be used as a proxy for socioeconomic status).*<br>*Provide clear definitions of the relevant terms used, how they were provided (by the participants/respondents, the researchers, or third parties), and the method(s) used to classify people into the different categories (e.g. self-report, census or administrative data, social media data, etc.)*<br>*Please provide details about how you controlled for confounding variables in your analyses.*
Population characteristics	*Describe the covariate-relevant population characteristics of the human research participants (e.g. age, genotypic information, past and current diagnosis and treatment categories). If you filled out the behavioural & social sciences study design questions and have nothing to add here, write "See above."*
Recruitment	*Describe how participants were recruited. Outline any potential self-selection bias or other biases that may be present and how these are likely to impact results.*
Ethics oversight	*Identify the organization(s) that approved the study protocol.*

Note that full information on the approval of the study protocol must also be provided in the manuscript.

Field-specific reporting

Please select the one below that is the best fit for your research. If you are not sure, read the appropriate sections before making your selection.

[x] Life sciences [ ] Behavioural & social sciences [ ] Ecological, evolutionary & environmental sciences

For a reference copy of the document with all sections, see nature.com/documents/nr-reporting-summary-flat.pdf

Life sciences study design

All studies must disclose on these points even when the disclosure is negative.

Sample size	No statistical test was used to determine sample size upfront. Sample size was determined based on observed effect sizes and standard errors from previous experiments, and according to previous knowledge of the variation of similar experimental setups. Data was checked for normality and similar variance between groups. Details for each experiment are included in the figure legends.
Data exclusions	No samples were excluded. Filters were applied for scRNAseq analysis to remove dead cells.
Replication	All experiments were replicated at least three times in biologically independent experiments to confirm reproducibility. All attempts to repeat the experiments were successful. Bulk RNAseq experiments were performed once using three technical replicates for each sample. ScRNAseq experiments were performed once, with a large number and good quality of cells which were sufficient to provide meaningful results. Details for each experiment are included in the figure legends.
Randomization	No specific method was used to randomize the experiments. For in vivo experiments, mice were allocated randomly to different groups before initiation of the treatments. For in vitro experiments, cells were divided equally to each group and treated with corresponding drug agents. Details for each experiment are included in the figure legends.
Blinding	Data collection was done blindly. Analysis were performed on data using unbiased approaches.

Reporting for specific materials, systems and methods

We require information from authors about some types of materials, experimental systems and methods used in many studies. Here, indicate whether each material, system or method listed is relevant to your study. If you are not sure if a list item applies to your research, read the appropriate section before selecting a response.

Materials & experimental systems

n/a	Involved in the study	
☐	☒	Antibodies
☐	☒	Eukaryotic cell lines
☒	☐	Palaeontology and archaeology
☐	☒	Animals and other organisms
☐	☒	Clinical data
☒	☐	Dual use research of concern
☒	☐	Plants

Methods

n/a	Involved in the study	
☒	☐	ChIP-seq
☐	☒	Flow cytometry
☒	☐	MRI-based neuroimaging

Antibodies

Antibodies used

Target Provider Reference Lot number Dilution
p-ERK1/2 Thr202/Tyr204 Cell Signaling #4370 (lot: 28) 1/1000
ERK Santa Cruz Biotechnology Sc-93 (lot:H2614) 1/2000
p-EGFR Tyr1068 Cell Signaling #2234 (lot:22) 1/2000
EGFR Cell Signaling #4267 (lot:24) 1/2000
N-cadherin Cell Signaling #4061 (lot:3) 1/500
p-RB Ser807/811 Cell Signaling #8516 (lot:8) 1/1000
RB Cell Signaling #9309 (lot:14) 1/2000
p27 Cell Signaling #3686 (lot:8) 1/1000
p53 Cell Signaling #48818 (lot:1) 1/1000
Cyclin D1 Cell Signaling #2978 (lot:13) 1/1000
Cyclin E1 Cell Signaling #20808 (lot:3) 1/1000
p-MLC2 Ser19 Cell Signaling #3671 (lot:7) 1/1000
MLC2 Cell Signaling #8505 (lot:6) 1/2000
PARP Cell Signaling #9542 (lot:15) 1/1000
Caspase-3 cleaved Cell Signaling #9661 (lot:47) 1/1000
Caspase-3 Cell Signaling #9662 (lot:19) 1/1000
HRAS Santa Cruz Biotechnology sc520 (lot:K207) 1/1000
FNTB Abclonal A2611 (lot:4000001923) 1/2000
ATF4 Cell Signaling #11815 (lot:6) 1/1000
CHOP Cell Signaling #2895 (lot:14) 1/1000
AGER Cell Signaling #55222 (lot:1) 1/1000
RhoA Santa Cruz Biotechnology Sc-418 (lot:J0914) 1/1000
RhoB ProteinTech 14326-1AP (lot:102742) 1/1000
RhoC Cell Signaling #3430 (lot:5) 1/5000

RhoE Cell Signaling #3664 (lot:1) 1/1000
LaminB1 Abcam ab16048 (lot:1022148-1) 1/2000
HDJ2 Abcam ab126774 (lot:GR82996-12) 1/1000
Actin Merck Millipore MAB1501 (lot:3845682) 1/50000
Tubulin Sigma T5168 (lot:84283) 1/50000

Validation

The following antibodies used in our study have been validated and detailed information could be found on the website from manufactures as listed below:
p-ERK1/2 Thr202/Tyr204, https://www.cellsignal.com/products/primary-antibodies/phospho-p44-42-mapk-erk1-2-thr202-tyr204-d13-14-4e-xp-rabbit-mab/4370
ERK, https://www.scbt.com/fr/p/erk-1-antibody-c-16
p-EGFR Tyr1068, https://www.cellsignal.com/products/primary-antibodies/phospho-egf-receptor-tyr1068-antibody/2234
EGFR, https://www.cellsignal.com/products/primary-antibodies/egf-receptor-d38b1-xp-rabbit-mab/4267
N-cadherin, https://www.cellsignal.com/products/primary-antibodies/n-cadherin-antibody/4061
p-RB Ser807/811, https://www.cellsignal.com/products/primary-antibodies/phospho-rb-ser807-811-d20b12-xp-rabbit-mab/8516
RB, https://www.cellsignal.com/products/primary-antibodies/rb-4h1-mouse-mab/9309
p27, https://www.cellsignal.com/products/primary-antibodies/p27-kip1-d69c12-xp-rabbit-mab/3686
p53, https://www.cellsignal.com/products/primary-antibodies/p53-do-7-mouse-mab/48818
Cyclin D1, https://www.cellsignal.com/products/primary-antibodies/cyclin-d1-92g2-rabbit-mab/2978
Cyclin E1, https://www.cellsignal.com/products/primary-antibodies/cyclin-e1-d7t3u-rabbit-mab/20808
p-MLC2 Ser19, https://www.cellsignal.com/products/primary-antibodies/phospho-myosin-light-chain-2-ser19-antibody/3671
MLC2, https://www.cellsignal.com/products/primary-antibodies/myosin-light-chain-2-d18e2-rabbit-mab/8505
PARP, https://www.cellsignal.com/products/primary-antibodies/parp-antibody/9542
Caspase-3 cleaved, https://www.cellsignal.com/products/primary-antibodies/cleaved-caspase-3-asp175-antibody/9661
Caspase-3, https://www.cellsignal.com/products/primary-antibodies/caspase-3-antibody/9662
HRAS, https://www.scbt.com/p/h-ras-antibody-c-20
FNTB, https://abclonal.com/catalog-antibodies/FNTBRabbitmAb/A2611#section2
ATF4, https://www.cellsignal.com/products/primary-antibodies/atf-4-d4b8-rabbit-mab/11815
CHOP, https://www.cellsignal.com/products/primary-antibodies/chop-l63f7-mouse-mab/2895
AGER, https://www.cellsignal.com/products/primary-antibodies/rage-e7i6s-rabbit-mab/55222
RhoA, https://www.scbt.com/fr/p/rho-a-antibody-26c4
RhoB, https://www.ptglab.com/fr/products/RHOB-Antibody-14326-1-AP.htm
RhoC, https://www.cellsignal.com/products/primary-antibodies/rhoc-d40e4-rabbit-mab/3430
RhoE, https://www.cellsignal.com/products/primary-antibodies/rhoe-4-mouse-mab/3664
LaminB1, https://www.abcam.com/en-fr/products/primary-antibodies/lamin-b1-antibody-nuclear-envelope-marker-ab16048
HDJ2, https://www.abcam.com/products/primary-antibodies/dnaja1-antibody-epr7248-ab126774.html
Actin, https://www.merckmillipore.com/FR/fr/product/Anti-Actin-Antibody-clone-C4,MM_NF-MAB1501
Tubulin, https://www.sigmaaldrich.com/FR/fr/product/sigma/t5168

Eukaryotic cell lines

Policy information about cell lines and Sex and Gender in Research

Cell line source(s)

The human NSCLC cell lines HCC4006 (CRL-2871, EGFRΔL747-E749, A750P), HCC827 (CRL-2868, EGFRΔE749-A750), HCC2935 (CRL-2869, EGFRΔE746-T751, S752I), Calu-1 (HTB-54, KRASG12C), H23 (CRL-5800, KRASG12C), and H3122 (EML4-ALK rearrangement) cell lines were obtained from the American Type Culture Collection (Manassas, VA, USA). The H3255 NSCLC cell line (EGFRL858R), the PC9 NSCLC cell line (EGFRdel E746-A750), the A375 cell line (CRL-1619, BRAFV600E), and the HCC364 BRAFV600E cell line, were a kind gift from Helene Blons (APHP, Paris, France), Antonio Maraver (IRCM, Montpellier, France), Nathalie Andrieu (CRCT, Toulouse, France), and David Santamaría (CIC, Salamanca, Spain), respectively.

Authentication

Authentication was performed using short tandem repeat DNA profiling. All the cells showed the expected morphology and growth features.

Mycoplasma contamination

All cell lines tested negative for mycoplasma. Tests were performed monthly.

Commonly misidentified lines
(See ICLAC register)

No commonly misidentified cell lines were used in this study

Animals and other research organisms

Policy information about studies involving animals; ARRIVE guidelines recommended for reporting animal research, and Sex and Gender in Research

Laboratory animals

Cell line xenograft experiments were performed in 6 to 8-week old female NMRI mice (Charles River Laboratories). The NSCLC PDX models EGFRT790M/L858R (TP103) and KRAS-G12C (TP60 and TP79) were generated in the Paz-Ares laboratory at the Instituto de Biomedicina de Sevilla (IBIS) and engrafted subcutaneously into the flank of 6 to 8-week old NSG mice. The NSCLC PDX model bearing EGFR exon 20 insertion (LU0387) have been described by Yang et al., Int J Cancer. 2016 (PMID: 26891175), and engrafted subcutaneously into the flank of 6 to 8-week old NSG mice.

Wild animals	none
Reporting on sex	both males and females have been included
Field-collected samples	none
Ethics oversight	All breeding, mouse husbandry, and in vivo experiments were performed with the approval of CREFRE ethical committee. All procedures involving animals and their care conformed to institutional guidelines for the use of animals in biomedical research. Animals were housed under controlled temperature and lighting (12/12-h light/dark cycle).

Note that full information on the approval of the study protocol must also be provided in the manuscript.

Clinical data

Policy information about clinical studies

All manuscripts should comply with the ICMJE guidelines for publication of clinical research and a completed CONSORT checklist must be included with all submissions.

Clinical trial registration	*Provide the trial registration number from ClinicalTrials.gov or an equivalent agency.*
Study protocol	*Note where the full trial protocol can be accessed OR if not available, explain why.*
Data collection	*Describe the settings and locales of data collection, noting the time periods of recruitment and data collection.*
Outcomes	*Describe how you pre-defined primary and secondary outcome measures and how you assessed these measures.*

Plants

Seed stocks	*Report on the source of all seed stocks or other plant material used. If applicable, state the seed stock centre and catalogue number. If plant specimens were collected from the field, describe the collection location, date and sampling procedures.*
Novel plant genotypes	*Describe the methods by which all novel plant genotypes were produced. This includes those generated by transgenic approaches, gene editing, chemical/radiation-based mutagenesis and hybridization. For transgenic lines, describe the transformation method, the number of independent lines analyzed and the generation upon which experiments were performed. For gene-edited lines, describe the editor used, the endogenous sequence targeted for editing, the targeting guide RNA sequence (if applicable) and how the editor was applied.*
Authentication	*Describe any authentication procedures for each seed stock used or novel genotype generated. Describe any experiments used to assess the effect of a mutation and, where applicable, how potential secondary effects (e.g. second site T-DNA insertions, mosiacism, off-target gene editing) were examined.*

Flow Cytometry

Plots

Confirm that:

- [x] The axis labels state the marker and fluorochrome used (e.g. CD4-FITC).
- [x] The axis scales are clearly visible. Include numbers along axes only for bottom left plot of group (a 'group' is an analysis of identical markers).
- [x] All plots are contour plots with outliers or pseudocolor plots.
- [x] A numerical value for number of cells or percentage (with statistics) is provided.

Methodology

Sample preparation	Cells were trypsinized and recovered in FACS buffer (0,04% BSA in PBS) and kept on ice. G1 (red) and S/G2 (green) cells were sorted at 4°C
Instrument	FACS Melody (BD Biosciences)
Software	BD FACSChorus v1.3.3
Cell population abundance	Purity and quality was high, based on sequencing and immunoblots. Color and general cell aspect of G1 (red) and S/G2 (green) cells was assessed by fluorescence microscopy after cell sorting
Gating strategy	Doublets and debris were excluded by gating on SSC/FSC, and live and singlet gates were used. A representative gating of FUCCI-labeled HCC4006 untreated and Osimertinib-treated cells is shown in Supplementary Figure 4A.

- [x] Tick this box to confirm that a figure exemplifying the gating strategy is provided in the Supplementary Information.
