# Supplementary material for: Farnesyltransferase inhibition overcomes oncogene-addicted non-small cell lung cancer adaptive resistance to targeted therapies
Source: Nat Commun. 2024 Jun 27;15:5345. doi: 10.1038/s41467-024-49360-4 (PMC11211478; doi:10.1038/s41467-024-49360-4)
Supplement: Supplementary file 4 — Description of Additional Supplementary File [file 41467_2024_49360_MOESM4_ESM.pdf]

Supplementary Data 1

Description: List of genes whose expression is correlated with *BPIFB1* (mucous/serous-related), *AGER* (AT1-related) and *SERPINE1* (EMT-related), and associated gene signatures (Hallmark, Reactome, Kegg). Pearson correlation coefficient >0.9. *p*-value was calculated using Pearson correlation test.

Supplementary Data 2

Description: Determination of shared gene signatures amongst seven different models of EGFR-mutant drug-tolerant cells. GSEA analysis was performed using transcriptomic data of seven models of EGFR-mutant drug-tolerant cells, and positively- or negatively-associated gene signatures (NOM *p*-value<0.05) were compared amongst the models. Nominal *p*-value and NES were calculated using GSEA software.

Supplementary Data 3

Description: Determination of drug-tolerant signature. Genes commonly upregulated (*i.e.* *p*<0.01, log2FC>0.5 in at least 6 out of 7 models; DTC_UP) and commonly downregulated (*i.e.* *p*<0.01, log2FC<-0.5 in at least 6 out of 7 models; DTC_DOWN). *p*-value was obtained by DESeq2 analysis.

Supplementary Data 4

Description: Gene signatures used in this study.

Supplementary Movie 1:

Description: FUCCI-transduced HCC827 subclone treated with 1 µM osimertinib. Cells were imaged with Incucyte S3 Live-Cell Analysis system (1 image/hour).

Supplementary Movie 2:

Description: FUCCI-transduced HCC4006 subclone treated with 1 µM osimertinib. Cells were imaged with Incucyte S3 Live-Cell Analysis system (1 image/hour).

Supplementary Movie 3:

Description: FUCCI-transduced PC9 subclone treated with 1 µM osimertinib. Cells were imaged with Incucyte S3 Live-Cell Analysis system (1 image/hour).

Supplementary Movie 4:

Description: FUCCI-transduced H3255 subclone treated with 1 µM osimertinib. Cells were imaged with Incucyte S3 Live-Cell Analysis system (1 image/hour).

Supplementary Movie 5:

Description: FUCCI-transduced HCC4006 subclone treated with 1 µM osimertinib (left) or 1 µM osimertinib in combination with 1 µM tipifarnib (right). Cells were imaged with Incucyte S3 Live-Cell Analysis system (1 image/hour).
